# Treatment response after 6 and 26 weeks is related to baseline glutamate and GABA levels in antipsychotic-naïve patients with psychosis

**DOI:** 10.1017/S0033291719002277

**Published:** 2020-10

**Authors:** Kirsten B. Bojesen, Bjørn H. Ebdrup, Kasper Jessen, Anne Sigvard, Karen Tangmose, Richard A.E. Edden, Henrik B.W. Larsson, Egill Rostrup, Brian V. Broberg, Birte Y. Glenthøj

**Affiliations:** 1 Center for Neuropsychiatric Schizophrenia Research (CNSR) & Center for Clinical Intervention and Neuropsychiatric Schizophrenia Research (CINS), Mental Health Centre Glostrup, University of Copenhagen, Glostrup, Denmark; 2 University of Copenhagen, Faculty of Health and Medical Sciences, Department of Clinical Medicine, Copenhagen, Denmark; 3 Russell H. Morgan Department of Radiology and Radiological Science, The Johns Hopkins University School of Medicine, F.M. Kirby Research Center for Functional Brain Imaging, Kennedy Krieger Institute, Baltimore, USA; 4 Functional Imaging Unit, Department of Clinical Physiology, Nuclear Medicine and PET, Rigshospitalet Glostrup, University of Copenhagen, Rigshospitalet, Glostrup, Denmark

**Keywords:** Anterior cingulate cortex, antipsychotic-naïve, first-episode psychosis, GABA, glutamate, magnetic resonance spectroscopy, thalamus, treatment outcome

## Abstract

**Background:**

Poor response to dopaminergic antipsychotics constitutes a major challenge in the treatment of psychotic disorders and markers for non-response during first-episode are warranted. Previous studies have found increased levels of glutamate and *γ*-aminobutyric acid (GABA) in non-responding first-episode patients compared to responders, but it is unknown if non-responders can be identified using reference levels from healthy controls (HCs).

**Methods:**

Thirty-nine antipsychotic-naïve patients with first-episode psychosis and 36 matched HCs underwent repeated assessments with the Positive and Negative Syndrome Scale and 3T magnetic resonance spectroscopy. Glutamate scaled to total creatine (/Cr) was measured in the anterior cingulate cortex (ACC) and left thalamus, and levels of GABA/Cr were measured in ACC. After 6 weeks, we re-examined 32 patients on aripiprazole monotherapy and 35 HCs, and after 26 weeks we re-examined 30 patients on naturalistic antipsychotic treatment and 32 HCs. The Andreasen criteria defined non-response.

**Results:**

Before treatment, thalamic glutamate/Cr was higher in the whole group of patients but levels normalized after treatment. ACC levels of glutamate/Cr and GABA/Cr were lower at all assessments and unaffected by treatment. When compared with HCs, non-responders at week 6 (19 patients) and week 26 (16 patients) had higher baseline glutamate/Cr in the thalamus. Moreover, non-responders at 26 weeks had lower baseline GABA/Cr in ACC. Baseline levels in responders and HCs did not differ.

**Conclusion:**

Glutamatergic and GABAergic abnormalities in antipsychotic-naïve patients appear driven by non-responders to antipsychotic treatment. If replicated, normative reference levels for glutamate and GABA may aid estimation of clinical prognosis in first-episode psychosis patients.

## Introduction

Insufficient response to antipsychotic medication is a major challenge in the treatment of psychotic disorders. Around one-third of patients are treatment resistant to the marketed dopaminergic antipsychotics (Cipriani *et al*., 2009), which all share an affinity for the striatal dopamine D2 receptors. Poor response to treatment in the chronic phase of schizophrenia has been associated with increased levels of glutamate in the anterior cingulate cortex (ACC) concurrent with relatively unaffected striatal dopamine function (Demjaha *et al*., 2014; Mouchlianitis *et al*., 2015). Recent findings suggest that glutamate and *γ*-aminobutyric acid (GABA) levels also are increased in antipsychotic-naïve or minimally treated non-responding patients when compared with levels in responders (de la Fuente-Sandoval *et al*., 2017; Egerton *et al*., 2018), whereas responding patients have higher striatal dopamine activity than non-responding patients (Wulff *et al*., 2015, 2019). These findings suggest the existence of a subgroup of non-responding patients with higher glutamate levels compared with responders. However, it is unknown if levels of glutamate and GABA in antipsychotic-naïve patients can serve as biomarkers for poor response to antipsychotic treatment compared with levels in healthy controls (HCs). Clinically, it would be highly relevant if non-responding patients could be identified already at illness onset by abnormal glutamate and GABA levels compared with reference levels in a healthy population. Moreover, it is unknown if baseline glutamate and GABA levels characterize non-responders beyond a treatment period of 4 weeks.

Preclinical data indicate that glutamatergic abnormalities originate from reduced activity of inhibitory GABAergic interneurons thereby causing either increased glutamate release through disinhibition of pyramidal neurons (Olney and Farber, 1995) or reduced release to maintain the excitatory-inhibitory balance (Lewis *et al*., 2012). This affects the cortico-striato-thalamo-cortical networks believed to be involved in the development of psychotic disorders (Carlsson *et al*., 1999). Especially abnormalities in dorsal ACC and the connected area thalamus are proposed to underlie schizophrenia (Williamson and Allman, 2012; Pratt *et al*., 2016). In support of this notion, *in vivo* studies of antipsychotic-naïve or minimally-treated patients with first-episode psychosis have reported aberrant levels of glutamatergic metabolites or GABA as measured with proton magnetic resonance spectroscopy (1H-MRS) in ACC and adjacent regions in terms of increased levels (Theberge *et al*., 2002, 2007; Bustillo *et al*., 2010; Kegeles *et al*., 2012; Yang *et al*., 2015; de la Fuente-Sandoval *et al*., 2017) or decreased levels (Wang *et al*., 2016; Chen *et al*., 2017), although no differences also have been reported (Aoyama *et al*., 2011). Increased glutamine levels have been found in the left thalamus (Theberge *et al*., 2002; Theberge *et al*., 2007) and thalamic glutamate levels are correlated with the genetic liability for schizophrenia spectrum disorders (Legind *et al*., 2019). Preclinical studies indicate a differential effect of specific antipsychotic compounds on glutamate and GABA levels (Bustillo *et al*., 2006; Yang and Wang, 2008; Xu *et al*., 2015), but currently, antipsychotic monotherapy in initially antipsychotic-naïve or minimally-treated patients has only been investigated in two longitudinal studies. Egerton *et al*. reported a reduction of glutamate levels in ACC and thalamus after 4 weeks of treatment with amisulpride (Egerton *et al*., 2018), and de la Fuente-Sandoval found reductions in the medial prefrontal cortex after 4 weeks of treatment with risperidone (de la Fuente-Sandoval *et al*., 2017). The effect of aripiprazole on glutamate levels is of particular interest since preclinical studies have suggested a neuroprotective effect of aripiprazole through reduction of glutamate release (Yang and Wang, 2008; Koprivica *et al*., 2011).

In this study, we examined glutamate levels in ACC and left thalamus and GABA levels in ACC at baseline and after 6 and 26 weeks in a sample of antipsychotic-naïve patients with first-episode psychosis and matched HCs. Patients were treated with aripiprazole monotherapy for the first 6 weeks but could be switched to another compound thereafter. First, we investigated the effect of 6 weeks aripiprazole monotherapy on glutamate and GABA levels. Next, we tested the primary hypothesis that baseline glutamate levels in the left thalamus and ACC would be increased and GABA levels in ACC would be decreased in non-responders after 6 and 26 weeks of treatment compared with levels in HCs.

## Patients and methods

### Participants and design

This longitudinal cohort study was initiated in January 2014 and carried out at mental health centers in the Capital Region of Denmark and conducted in accordance with the Declaration of Helsinki and approved by the Committee on Biomedical Research Ethics (H-3-2013-149). All participants provided written informed consent after the study procedures had been explained. We included legally competent patients fulfilling a diagnosis of International Classification of Diseases, 10^th^ revision (ICD-10) criteria for schizophrenia, schizoaffective disorder, or non-organic psychosis as determined with the Schedules for Clinical Assessment in Neuropsychiatry (SCAN) (Wing *et al*., 1990). Inclusion criteria were: age 18–45 years, no lifetime exposure to antipsychotics or central nervous system stimulants (verified by medical record), and no substance abuse (ICD-10 F1x.2) in the previous 3 months. Tobacco use and current occasional substance use (ICD-10 F1x.1) were accepted and assessed through self-report and a urine drug test (Rapid Response, Jepsen HealthCare, Tune, DK). Benzodiazepines were allowed on per needed basis (maximum three times per day) to reduce anxiety and secure sleep, although no later than 12 h prior to examinations. HCs were recruited through online advertisement (www.forsøgsperson.dk) and matched on age, sex, and parental socioeconomic status. Further details of the in- and exclusion criteria are provided in online Supplementary Methods.

All participants underwent clinical assessment and magnetic resonance imaging (MRI) at baseline and at week 6 and 26. Patients were treated with aripiprazole monotherapy (doses of 5–30 mg/day, tool compound) for the first 6 weeks and the use of other psychoactive compounds (antipsychotics, antidepressants, or mood stabilizers) was not allowed. After 6 weeks patients were allowed to switch to another antipsychotic compound if the clinical effect was inadequate or side-effects outweighed the clinical effect. HCs were not treated. Patients, who prematurely terminated aripiprazole treatment, were not re-examined at 6 weeks, but re-invited for examination at 26 weeks. A scanner upgrade lasting 8 months resulted in some missing MRI examinations as reported in Table 1. Details of participants scanned before and after the upgrade are provided in online Supplementary Methods.

**Table 1.** Demographics and clinical characteristics at baseline and at 6 and 26 weeks

	Baseline		6 weeks		26 weeks		Statistics
	FEP	HCs	FEP_6 weeks_	HCs_6 weeks_	FEP_26 weeks_	HCs_26 weeks_	
Number of participants (M/F)	44 (21/23)	36 (18/18)	32 (15/17)	35 (17/18)	30 (17/13)	32 (16/16)	
MRI data (*N*)	39	36	31	35	26	31	
Age ± s.d., years	23.0 ± 5.4	22.7 ± 4.4					FEP *v*. HC_Baseline_: *T*(78) = 0.32, *p* = 0.75
Parental socioeconomic status (high/moderate/low/missing)	13/25/5/1	9/21/5/1					FEP *v*. HC_Baseline_: Fisher's *p* = 0.90
Education ± s.d., years	11.6 ± 2.0	13.9 ± 2.1					FEP *v*. HC_Baseline_: *T*(78) = −5.11, P < 0.001
Handedness, (right/left/ambidextrous)	33/6/4	30/5/1					FEP *v*. HC_Baseline_: Fisher's *p* = 0.63
Duration of untreated psychosis, Median (25–75^th^ percentile) in weeks	58.0 (25.0−178.0)						
Ethnicity (White/Asian/African/other)	41/2/1/0						
Current tobacco use, yes/no	18/26	3/33	15/17	4/31	19/11	3/29	FEP *v*. HC_Baseline_: Fisher's *p* = 0.001 FEP *v*. HC_6 weeks_: Fisher's *p* = 0.002 FEP *v*. HC_26 weeks_: Fisher's *p* < 0.001
Current cannabis use, yes/no	5/39	2/34	32/0	35/0	2/28	0/34	FEP *v*. HC_Baseline_: Fisher's *p* = 0.45 FEP *v*. HC_26 weeks_: Fisher's *p* = 0.22
PANSS, mean ± s.d. (% decrease from baseline ± s.d.)^c^							
Total	73.2 ± 14.1	−	56.6 ± 12.3*** (26.6 ± 12.3%)	−	58.2 ± 13.2*** (28.2 ± 13.2%)	−	FEP baseline *v*. 6 and 26 weeks: *F*(2,27.5) = 28.39, *p* < 0.001^a^
Positive	17.9 ± 4.4	−	12.6 ± 4.4*** (46.6 ± 37.1%)	−	12.3 ± 3.9*** (51.9 ± 33.5%)	−	FEP baseline *v*. 6 and 26 weeks: *F*(2,31.5) = 26.99, *p* < 0.001^a^
Negative	18.8 ± 5.2	−	15.9 ± 4.7** (7.4 ± 79.4%)	−	17.6 ± 6.0 (1.9 ± 59.4%)	−	FEP baseline *v*. 6 and 26 weeks: *F*(2,27.5) = 28.39, *p* = 0.02^a^
General	36.4 ± 7.6	−	28.1 ± 6.6*** (32.2 ± 37.4%)	−	28.1 ± 6.1*** (40.7 ± 24.6%)	−	FEP baseline *v*. 6 and 26 weeks: *F*(2,29.3) = 32.77, *p* < 0.001^a^
Clinical Global Impression, ±s.d., (Improvement ± s.d.)	4.3 ± 0.6 (−)	−	3.6 ± 0.6*** (2.3 ± 0.6)	−	3.6 ± 0.6*** (2.4 ± 0.9)	−	FEP baseline *v*. 6 and 26 weeks: *F*(2,30.9) = 22.26, *p* < 0.001^a^
PSP score, mean ± s.d.	45.6 ± 13.4	89.7 ± 3.7	57.0 ± 11.7***	90.1 ± 4.0	54.3 ± 11.5***	89.1 ± 4.5	FEP *v*. HC (group×time interaction)^b^: *F*(2,61.2) = 10.09, *p* < 0.001
Benzodiazepines (daily/per need/no)	1/6/37	0/0/36	2/1/29	0/0/35	0/2/28	0/0/32	FEP *v*. HC_Baseline_: Fisher's *p* = 0.03 FEP *v*. HC_6 weeks_: Fisher's *p* = 0.10 FEP *v*. HC_26 weeks_: Fisher's *p* = 0.23
Aripiprazole dose in mg, Median (25–75^th^ percentile)	−	−	10 (5–15)	−	−	−	
Serum aripiprazole concentration in *μ*g/L, Median (25–75^th^ percentile)			253.0 (146.0–363.0)				
Antipsychotic treatment (None/Ari/Que/Ris/Pal/Ola/Clo)	−	−	0/32/0/0/0/0/0	−	2/20/3/2/1/1/1	−	

FEP, first-episode psychosis patients; HCs, healthy controls; MRI, magnetic resonance imaging; S.D., standard deviation; PANSS, Positive and Negative Syndrome Scale; PSP, Personal and Social Performance scale; Ari, aripiprazole; Que, quetiapine; Ris, risperidone; Pal, paliperidone; Ola, olanzapine; Clo, clozapine.

a Main effect of time.

b Significant interaction due to an increase in the FEP group after 6 and 26 weeks compared with baseline but not in the HC group.

c For the PANSS reduction %, the minimum score was initially subtracted. ***p < 0.001 in post hoc tests.

### Clinical assessments

In patients, psychopathology was assessed by trained raters using the Positive and Negative Syndrome Scale (PANSS) (Kay *et al*., 1987), and percentage change in PANSS scores was calculated after subtraction of the minimum score (Leucht *et al*., 2007). Symptom severity was assessed with the Clinical Global Impression (CGI) scale (Guy, 1976), and side effects with the short version of the Task Force for Clinical Investigation (*Udvalget for Kliniske Undersøgelser*) (UKU) (Lingjaerde *et al*., 1987). Level of functioning was assessed both in patients and HCs using the Personal and Social Performance Scale (PSP) (Morosini *et al*., 2000). Clinical response after 6 and 26 weeks of treatment was assessed using the Andreasen remission criteria (Andreasen *et al*., 2005) (described in the online Supplementary Methods) with the modification that remission status was based on PANSS scores during the previous week rather than 6 months. The terms responder and non-responder are therefore used instead of remitter and non-remitter.

### Magnetic resonance spectroscopy

All scans were performed on a 3.0 Tesla Philips scanner with a 32-channel head coil as described previously in (Bojesen *et al*., 2018) and in the online Supplementary Methods together with details of the T1-weighted structural image acquisition. Levels of glutamate and other major metabolites were estimated using the PRESS sequence (TR 3000 ms, TE 30 ms, 128 averages, MOIST water-suppression) with one 2.0 × 2.0 × 2.0 cm voxel in ACC (Brodmann area 24 and 32) and one 2.0 × 1.5 × 2.0 cm voxel in the left thalamus. Levels of GABA + macromolecules and glutamate + glutamine (glx) were estimated in a 3.0 × 3.0 × 3.0 cm voxel in ACC using the MEGAPRESS sequence (Mescher *et al*., 1998) (TE = 68 ms; TR = 2000 ms, 14 ms editing pulse applied at 1.9 ppm and 7.5 ppm, 320 averages, MOIST water suppression). LCModel version 6.3-1J (Provencher, 1993) was used to analyze the PRESS acquisitions fitted in the spectral range between 0.2 and 4.0 ppm, as previously described (Bojesen *et al*., 2018). Gannet version 2.1 (Edden *et al*., 2014) was used to analyze the MEGAPRESS acquisitions fitted in the spectral range between 2.79 and 3.55 ppm using water scaling to get an estimate of GABA and total creatine + phosphocreatine (creatine) levels. Spectral quality was initially assessed by visual inspection. In accepted spectra, the exclusion criteria for individual metabolites were Cramér–Rao lower bound (CRLB) >20% for PRESS acquisitions and Gannet signal fit error >15% for MEGAPRESS acquisitions. Details on 1H-MRS and data quality are provided in the online Supplementary Methods, online Supplementary Results, and Fig. S1. We used glutamate and GABA as a ratio to total creatine (/Cr) as the primary outcome measures since the study was inspired by the first cross-sectional study of first-episode patients that reported an association between glutamate/Cr and treatment outcomes (Egerton *et al*., 2012). To allow for comparisons with other studies, neurotransmitter levels in institutional units (IU) were also calculated as described in the online Supplementary Methods. Findings in metabolite levels in IU are mentioned in the Result section and statistics are reported in the online Supplementary Results.

### Statistical analyses

The significance level for the planned main outcomes baseline glutamate/Cr and GABA/Cr in relation to treatment outcome after 6 and 26 weeks was defined as *p* < 0.05 in the analyses. The primary hypothesis that baseline levels of glutamate and GABA (dependent variables) would differ in non-responders after 6 and 26 weeks compared with baseline levels in HCs was tested with a one-way analysis of variance (ANOVA) and the significance level was adjusted for 2 comparisons (both responders and non-responders were compared with HCs, *p* < 0.05/2 = 0.025) and with adjustment for age and sex. Next, binary logistic regression was applied to explore if baseline glutamate and GABA levels could predict non-response compared with reference values in HCs. In secondary analyses, the effect of treatment on glutamate and GABA levels was analyzed in separate linear mixed models with the independent variables group (patients *v.* HCs) and time (0, 6, and 26 weeks). The group×time interaction expressed if the trajectory of glutamate and GABA levels was different in patients and controls. If the interaction was not significant, it was removed from the model. We adjusted for the following factors, which might influence longitudinal glutamate and GABA levels: sex (O'Gorman *et al*., 2011), age (Marsman *et al*., 2013; Rowland *et al*., 2016), tobacco use, cannabis use, and scanner upgrade. Group differences in demographical- and clinical variables, as well as spectral quality, were tested with χ^2^, Fisher's exact test, or linear mixed models, as appropriate. In explorative analyses, the associations between baseline glutamate or GABA levels and reduction of PANSS total and positive scores were investigated with Spearman or Pearson's correlations. Statistical analyses were performed using SAS version 7.1 (SAS Institute, Cary, NC, USA).

## Results

### Participants

Forty-four initially antipsychotic-naïve first-episode patients with psychosis and 36 HCs were recruited. Clinical characteristics are summarized in Table 1 for all patients and HCs. Groups did not differ on age, parental socioeconomic status, current cannabis use, or handedness, but patients had fewer years of education, lower level of functioning, and more were smokers. After 6 and 26 weeks of treatment, patients' functioning level significantly increased, and all PANSS scores significantly decreased, except for PANSS negative after 26 weeks. After 6 weeks, 12 of 44 patients and 1 of 36 HCs were not re-assessed; and after 26 weeks, 11 of 44 patients and 2 of 36 HCs were not re-assessed. Twenty-four patients completed re-examinations at both week 6 and 26. Reasons for drop-out and comparisons between completers and non-completers are provided in online Supplementary Methods.

Clinical characteristics and statistics for responders and non-responders with a baseline MRI scan are summarized in Table 2. Briefly, responders and non-responders did not differ in age, smoking status, cannabis use, or baseline PANSS scores. Non-responders at 6 weeks received significantly higher aripiprazole doses, but serum aripiprazole levels did not significantly differ compared with responders. Three responders and five non-responders at 6 weeks changed their response status after 26 weeks. Five patients were switched to another antipsychotic compound at 6 weeks and one of these responded at 26 weeks, whereas four remained non-responders.

**Table 2:** Demographics of responders and non-responders at 6 and 26 weeks

	Non-responders 6 weeks	Responders 6 weeks	Statistics NR *v*. R 6 weeks	Non-responders 26 weeks	Responders 26 weeks	Statistics NR *v*. R 26 weeks
*N*^a^	19	11		16	9	
Age ± s.d., years	23.3 ± 6.3	21.8 ± 3.2	*T*(28) = 0.47, *p* = 0.40	22.7 ± 5.7	21.8 ± 3.3	T(23) = −0.44 *p* = 0.67
Tobacco use baseline, yes/no	8/11	4/7	Fisher's *p* = 0.29	9/7	4/5	Fisher's *p* = 0.69
Cannabis use baseline, yes/no	1/18	1/10	Fisher's *p* = 1.00	2/14	0/9	Fisher's *p* = 0.52
PANSS baseline, median (25–75^th^ percentile)						
Total	73.0 (66.0–85.0)	69.0 (55.0–76.0)	Z = −1.33, *p* = 0.18	78.5 (71.5–83.0)	69.0 (58.0–83.0)	Z = −0.62, *p* = 0.53
Positive	19.0 (14.0–20.0)	16.0 (13.0–18.0)	Z = −0.99, *p* = 0.32	18.0 (14.5–19.5)	19.0 (17.0–20.0)	Z = 0.80, *p* = 0.43
Negative	21.0 (18.0–23.0)	17.0 (13.0–21.0)	Z = −1.69, *p* = 0.09	21.5 (19.0–23.0)	17.0 (14.0–22.0)	Z = −1.31, *p* = 0.19
General	34.0 (30.0–42.0)	32.0 (28.0–39.0)	Z = −0.99, *p* = 0.32	38.5 (32.0–42.0)	32.0 (28.0–42.0)	Z = −0.94, *p* = 0.35
Aripiprazole, median (25–75^th^ percentile)	15 mg (5–15 mg)	10 mg (5–10 mg)	Z = −2.05, *p* = 0.04	−	−	−
Serum aripiprazole, median (25–75^th^ percentile)	116.1 *µ*g/L (63.7–225.1 *µ*g/L)	83.8 *µ*g/L (65.5–136.8 *µ*g/L)	Z = −0.73, *p* = 0.46	−	−	−

NR, non-responders; R, responders.

a Only patients with an MRI scan at baseline are included.

### Treatment effects on glutamate and GABA levels

#### Glutamate levels in the left thalamus

**Fig. 1.**
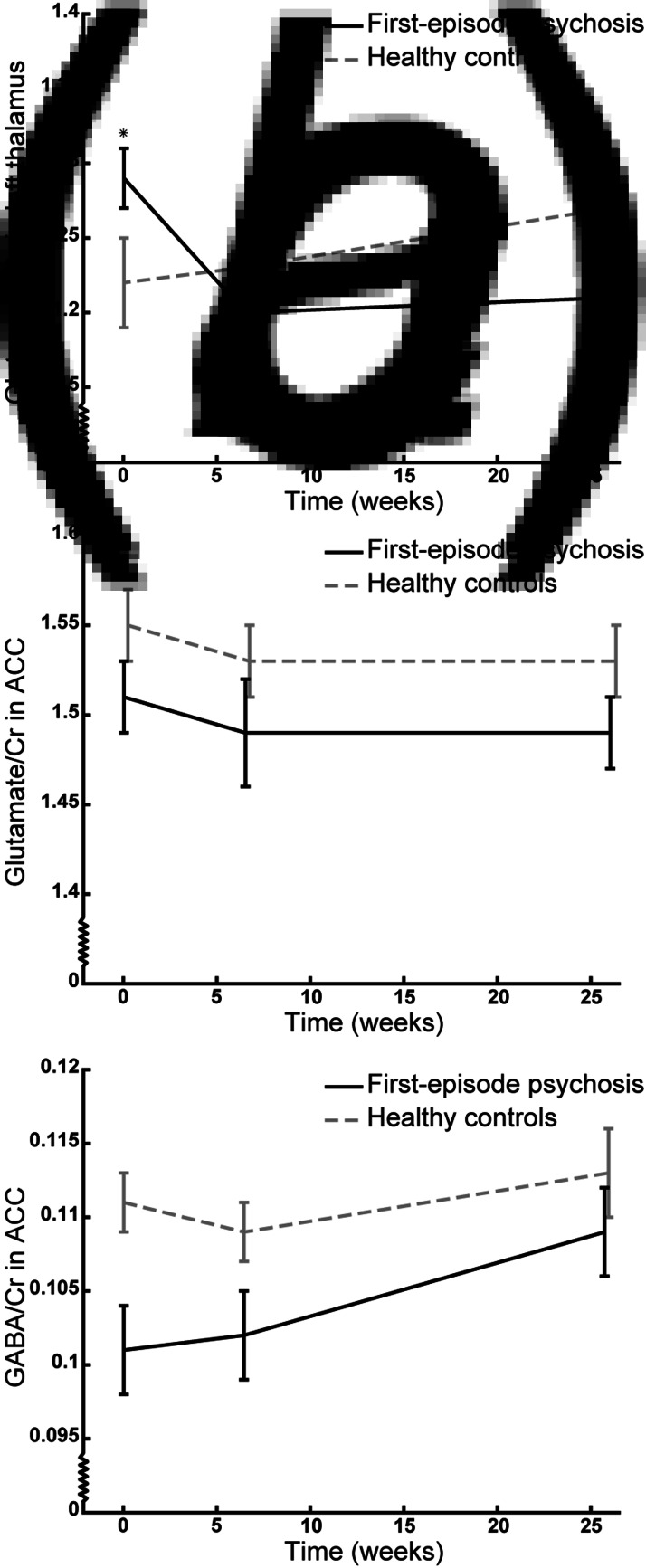
The trajectory of mean glutamate/Cr levels in the left thalamus (*a*) and anterior cingulate cortex (ACC) (*b*) and GABA/Cr levels in ACC (*c*) of initially antipsychotic-naïve patients with first-episode psychosis (black line) and healthy controls (grey line) at baseline, and after 6 weeks monotherapy with aripiprazole, and 26 weeks of naturalistic antipsychotic treatment. The vertical bars represent standard errors. *p < 0.05 in post hoc testing. Note that the *Y*-axis does not origin in 0;0.

The trajectory of glutamate/Cr levels in the left thalamus was different in patients and HCs (significant group×time interaction: *F*_(2,61.5)_ = 5.30, *p* = 0.008) indicating an effect of treatment. Post hoc tests revealed increased glutamate/Cr levels in patients at baseline compared with HCs [*T*_(70.6)_ = 2.11, *p* = 0.04, estimate = 0.08 (CI 0.004–0.15)], whereas there were no differences between groups after 6 weeks of treatment with aripiprazole (*p* = 0.55) and 26 weeks of naturalistic antipsychotic treatment (*p* = 0.08) (Fig. 1*a*). The interaction remained significant after adjusting for age, sex, smoking status, cannabis use, and scanner upgrade (*p* values: 0.005–0.008).

A similar trend was found for glutamate levels in IU in the thalamus, although not statistically significant.

#### Glutamate levels in ACC

The trajectory of glutamate/Cr levels in ACC did not differ between patients and HCs (group×time insignificant: *p* = 0.75) indicating no effect of treatment. The patients had lower levels of glutamate/Cr at all examinations compared with HCs [significant main effect of group: *F*_(1,66.8)_ = 4.10, *p* = 0.047, estimate = −0.04 (CI −0.08 to −0.001), Fig. 1*b*] and this remained significant when removing the group×time interaction (*p* = 0.046) and adjusting for age (*p* = 0.02) and cannabis use (*p* = 0.04) but not when adjusting for sex, smoking status, and scanner upgrade (*p* = 0.05–0.10). There was no main effect of time.

For glutamate levels in IU in ACC, the interaction and main effect of the group were insignificant.

#### GABA levels in ACC

The trajectory of GABA/Cr levels in ACC did not differ between patients and HCs (group×time insignificant, *p* = 0.62) indicating no effect of treatment. The patients had lower levels of GABA/Cr at all examinations compared with HCs [significant main effect of group: *F*_(1,74.2)_ = 9.16, *p* = 0.003, estimate = −0.008 (CI −0.01 to −0.003), Fig. 1*c*] and this was also significant when removing the group×time interaction (*p* = 0.001) and adjusting for age, sex, smoking status, cannabis use, and scanner upgrade and excluding subjects that used benzodiazepines either occasionally or daily (*p* = 0.001–0.03). There was no main effect of time.

Similar results were found for GABA levels in IU in ACC, although the main effect of the group only was borderline significant.

The mean levels of other metabolites at all assessments are summarized in the online Supplementary Tables S8–S10 for creatine scaled values, levels in IU, and water scaled values, respectively.

### Baseline glutamate and GABA levels in non-responders and responders compared with healthy controls

The baseline levels of metabolites in non-responders, responders, and HCs at 6 and 26 weeks are summarized in Table 3 for creatine scaled values, Table S11 for levels in IU, and Table S12 for water scaled values.

**Table 3:** Baseline neurometabolite levels scaled to total creatine in responders and non-responders after 6 and 26 weeks compared with HCs

	Non-responders 6 weeks	Responders 6 weeks	HCs	*p*_6 weeks_^E^ NR *v*. HCs	*p*_6 weeks_^E^ R *v*. HCs	Non-responders 26 weeks	Responders 26 weeks	HCs	*p*_26 weeks_^E^ NR *v*. HCs	*p*_26 weeks_^E^ R *v*. HCs
	Mean ± s.d., (*n*^b^)	Mean ± s.d., (*n*^b^)	Mean ± s.d., (*n*^b^)			Mean ± s.d., (*n*^b^)	Mean ± s.d., (*n*^b^)	Mean ± s.d., (*n*^b^)		
*Thalamus baseline levels*										
Glutamate/Cr	1.32 ± 0.15, (18)	1.26 ± 0.15, (11)	1.22 ± 0.15, (31)	*p* = 0.007	*p* = 0.51	1.33 ± 0.14, (15)	1.34 ± 0.17, (9)	1.22 ± 0.14, (28)	*p* = 0.017	*p* = 0.09
Glx/Cr	1.79 ± 0.28, (18)	1.76 ± 0.26, (11)	1.77 ± 0.22, (32)	*p* = 0.59	*p* = 0.88	1.89 ± 0.27, (15)	1.88 ± 0.31, (9)	1.77 ± 0.22, (29)	*p* = 0.18	*p* = 0.40
NAA/Cr	1.33 ± 0.15, (18)	1.25 ± 0.11, (11)	1.30 ± 0.09, (32)	*p* = 0.27	*p* = 0.14	1.29 ± 0.14, (15)	1.32 ± 0.19, (9)	1.31 ± 0.09, (29)	*p* = 0.59	*p* = 0.84
Myo-inositol/Cr	0.66 ± 0.10, (18)	0.62 ± 0.09, (11)	0.66 ± 0.08, (32)	*p* = 0.87	*p* = 0.16	0.64 ± 0.10, (15)	0.67 ± 0.11 , (9)	0.67 ± 0.08, (29)	*p* = 0.25	*p* = 0.91
Choline/Cr	0.30 ± 0.04, (18)	0.30 ± 0.02, (11)	0.31 ± 0.03, (32)	*p* = 0.34	*p* = 0.17	0.30 ± 0.03, (15)	0.30 ± 0.04, (9)	0.31 ± 0.03, (29)	*p* = 0.25	*p* = 0.13
PCr + Cr^a^	5.33 ± 0.62, (18)	5.66 ± 0.40, (11)	5.60 ± 0.31, (32)	*p* = 0.02	*p* = 0.53	5.47 ± 0.59, (15)	5.45 ± 0.76, (9)	5.59 ± 0.31, (29)	*p* = 0.39	*p* = 0.66
Grey matter (%)	20.7 ± 6.5, (18)	17.8 ± 7.2, (11)	19.3 ± 4,6 (32)	*p* = 0.33	*p* = 0.37	19.5 ± 9.0, (15)	18.4 ± 5.8, (9)	19.4 ± 4.6, (29)	*p* = 0.87	*p* = 0.65
White matter (%)	78.3 ± 8.9, (18)	82.1 ± 7.3, (11)	80.6 ± 4.6, (32)	*p* = 0.18	*p* = 0.44	78.6 ± 10.7, (15)	81.5 ± 5.8, (9)	80.5 ± 4.6, (29)	*p* = 0.36	*p* = 0.68
*ACC*^c^ *baseline levels*										
Glutamate/Cr	1.48 ± 0.19, (17)	1.55 ± 0.08, (11)	1.54 ± 0.10, (35)	*p* = 0.11	*p* = 0.79	1.52 ± 0.13, (15)	1.54 ± 0.12, (8)	1.55 ± 0.10, (32)	*p* = 0.37	*p* = 0.78
Glx/Cr	1.95 ± 0.27, (17)	2.06 ± 0.18, (11)	2.02 ± 0.18, (35)	*p* = 0.55	*p* = 0.93	2.02 ± 0.16, (15)	2.05 ± 0.24, (8)	2.02 ± 0.18, (32)	*p* = 0.95	*p* = 0.80
Gln/Cr	0.50 ± 0.12, (15)	0.56 ± 0.12, (8)	0.53 ± 0.08, (26)	*p* = 0.84	*p* = 0.53	0.51 ± 0.11, (15)	0.53 ± 0.13, (7)	0.52 ± 0.08, (23)	*p* = 0.95	*p* = 0.80
NAA/Cr	1.22 ± 0.06, (17)	1.22 ± 0.05, (11)	1.26 ± 0.06, (35)	*p* = 0.01	*p* = 0.09	1.21 ± 0.06, (15)	1.23 ± 0.06, (8)	1.26 ± 0.06, (32)	*p* = 0.01	*p* = 0.20
Myo-inositol/Cr	0.83 ± 0.07, (17)	0.83 ± 0.06, (11)	0.85 ± 0.07, (35)	*p* = 0.33	*p* = 0.46	0.81 ± 0.07, (15)	0.86 ± 0.07, (8)	0.85 ± 0.07, (32)	*p* = 0.04	*p* = 0.74
Choline/Cr	0.27 ± 0.03, (17)	0.28 ± 0.03, (11)	0.29 ± 0.03, (35)	*p* = 0.02	*p* = 0.13	0.27 ± 0.03, (15)	0.28 ± 0.03, (8)	0.29 ± 0.03, (32)	*p* = 0.006	*p* = 0.29
PCr + Cr^a^	5.31 ± 0.28, (17)	5.39 ± 0.19, (11)	5.28 ± 0.25, (35)	*p* = 0.74	*p* = 0.16	5.31 ± 0.27, (15)	5.31 ± 0.19, (8)	5.26 ± 0.25, (32)	*p* = 0.43	*p* = 0.47
Grey matter (%)	67.5 ± 6.8, (17)	69.4 ± 4.5, (11)	68.0 ± 3.9, (35)	*p* = 0.73	*p* = 0.42	65.2 ± 5.9, (15)	70.9 ± 8.0, (8)	67.3 ± 3.4, (32)	*p* = 0.18	*p* = 0.09
White matter (%)	13.4 ± 3.5, (17)	15.1 ± 5.5, (11)	13.0 ± 2.6, (35)	*p* = 0.85	*p* = 0.03	14.5 ± 5.0, (15)	14.0 ± 4.0, (8)	13.0 ± 2.7, (32)	*p* = 0.15	*p* = 0.36
*ACC*^d^ *baseline levels*										
GABA/Cr	0.102 ± 0.012, (11)	0.102 ± 0.019, (9)	0.111 ± 0.011, (31)	*p* = 0.04	*p* = 0.08	0.100 ± 0.013, (8)	0.109 ± 0.008, (6)	0.111 ± 0.011, (28)	*p* = 0.008	*p* = 0.80
Glx/Cr	0.114 ± 0.016, (11)	0.107 ±, 0.014 (9)	0.118 ± 0.011, (31)	*p* = 0.006	*p* = 0.009	0.113 ± 0.010, (8)	0.108 ± 0.013, (6)	0.118 ± 0.011, (28)	*p* = 0.25	*p* = 0.03
PCr + Cr^a^	11.21 ± 0.51, (11)	10.66 ± 1.16, (9)	10.90 ± 0.66, (31)	*p* = 0.41	*p* = 0.57	11.05 ± 0.52, (8)	10.38 ± 1.23, (6)	10.88 ± 0.63, (28)	*p* = 0.44	*p* = 0.20
Grey matter (%)	51.0 ± 3.1, (11)	53.7 ± 3.6, (9)	53.1 ± 3.2 , (31)	*p* = 0.08	*p* = 0.69	48.9 ± 5.4, (8)	53.3 ± 3.8, (6)	52.7 ± 2.9, (28)	*p* = 0.02	*p* = 0.88
White matter (%)	31.9 ± 2.4, (11)	32.5 ± 5.1, (9)	30.5 ± 3.1, (31)	*p* = 0.36	*p* = 0.09	32.9 ± 3.8, (8)	32.3 ± 5.2, (6)	30.6 ± 3.2, (28)	*p* = 0.11	*p* = 0.22

NR, non-responders; R, responders; HCs, healthy controls; ACC, anterior cingulate cortex; Glx, glutamate + glutamine; Gln, glutamine; NAA, *N*-acetyl aspartate; PCr + Cr, total creatine (phosphocreatine + creatine); GABA, *γ*-aminobutyric acid.

a Water-scaled value of PCr + Cr provided by LCModel and Gannet and used as a reference in metabolite/Cr values.

b *N* is the number of spectra analyzed.

c Metabolites in a 2.0 × 2.0 × 2.0 cm voxel.

d Metabolites in a 3.0 × 3.0 × 3.0 cm voxel (details provided in the patients and methods section).

e Adjusted for age and sex.

#### Baseline glutamate levels in the left thalamus

**Fig. 2.**
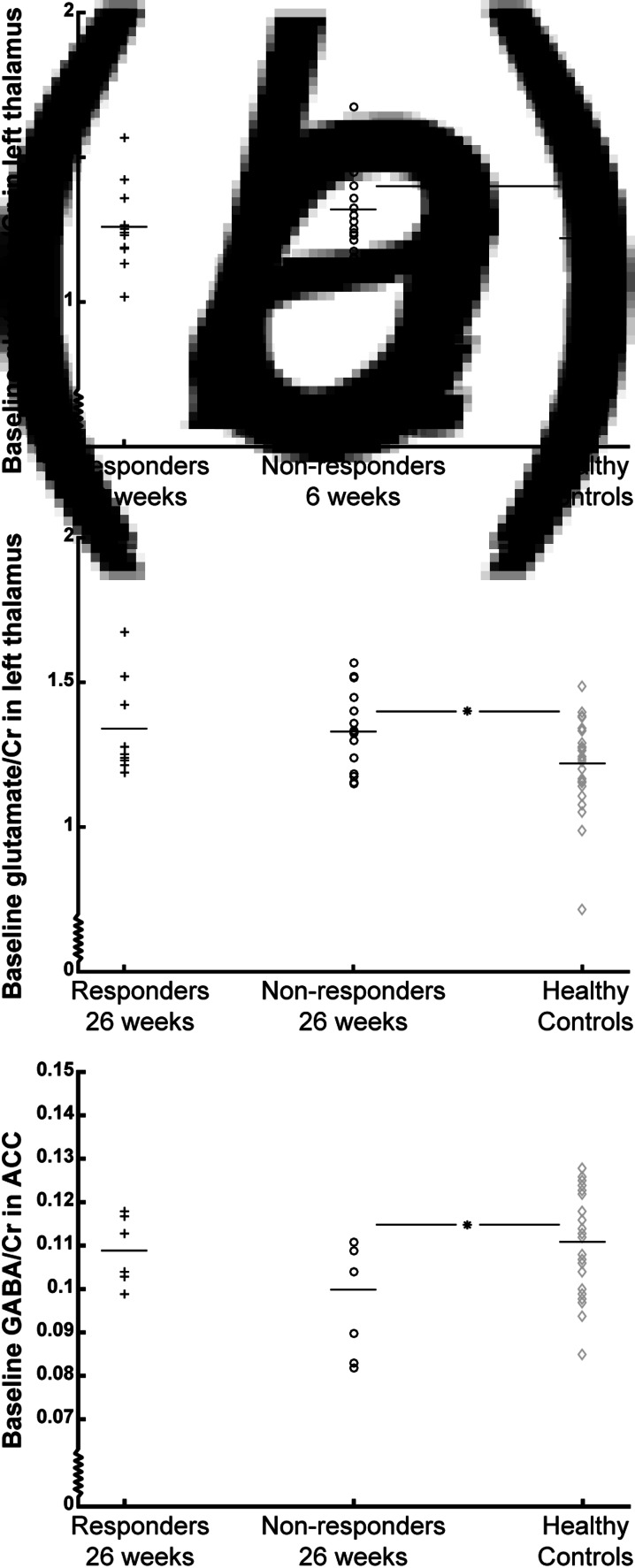
Baseline levels of glutamate/Cr in the thalamus and GABA/Cr in the anterior cingulate cortex (ACC) of responders (black plusses), non-responders (black circles), and healthy controls (grey diamonds). Mean values are represented by horizontal bars. Baseline glutamate/Cr levels in the left thalamus are shown according to treatment response after 6 weeks (A) and 26 weeks (B), and baseline GABA/Cr levels are shown according to treatment response after 26 weeks (C). *p < 0.025. Note that the *Y*-axis does not origin in 0;0.

Baseline glutamate/Cr levels were higher in non-responders at 6 weeks compared with baseline levels in HCs [overall model *F*_(2,57)_ = 2.83, *p* = 0.067, after adjustment for age and sex: overall model *F*_(2,55)_ = 2.89, *p* = 0.03; post hoc test: *T*_(55)_ = 2.82, *p* = 0.007], whereas responders and HCs did not differ (*p* = 0.51) (Fig. 2*a*). Similarly, non-responders at 26 weeks had higher baseline levels [overall model *F*_(2,49)_ = 3.59, *p* = 0.04; post hoc test: *T*_(49)_ = 2.34, *p* = 0.023; after adjustment for age and sex: overall model *F*_(2,47)_ = 3.89, *p* = 0.008; post hoc test: *T*_(47)_ = 2.48, *p* = 0.017], whereas responders and HCs did not differ (*p* = 0.05, after adjustment for age and sex: *p* = 0.09) (Fig. 2*b*). For glutamate IU, a similar result was found for non-responders at 26 weeks, but not at 6 weeks.

#### Baseline glutamate levels in ACC

There were no differences between baseline glutamate/Cr levels in neither non-responders nor responders at 6 and 26 weeks compared with HCs [6 weeks: overall model *p* = 0.18, after adjustment for age and sex: *F*_(2,58)_ = 3.60, *p* = 0.01; post hoc tests: *p*_Non-responders *v.* HCs_ = 0.11 and *p*_Responders *v.* HCs_ = 0.79; 26 weeks: overall model *p* = 0.69, after adjustment for age and sex: *p* = 0.60]. Similar results were found for glutamate IU.

#### Baseline GABA levels in ACC

There was no difference in baseline GABA/Cr levels in non-responders at 6 weeks compared with HCs (overall model: *p* = 0.08, after adjustment for age and sex: *p* = 0.09), but non-responders at 26 weeks had significantly lower baseline GABA/Cr levels compared with HCs [overall model: *F*_(2,39)_ = 3.10, *p* = 0.056, after adjustment for age and sex: overall model: *F*_(2,37)_ = 2.64, *p* = 0.049; post hoc test: *T*_(37)_ = −2.78, *p* = 0.008], whereas responders and HCs did not differ (*p* = 0.79) (Fig. 2*c*). For GABA IU, the associations were not significant.

#### Baseline levels of other neurometabolites in the left thalamus and ACC

In the left thalamus, significantly lower baseline levels of total creatine were observed in patients not responding at 6 weeks (*p* = 0.02). In the 2.0×2.0×2.0 cm ACC PRESS voxel, non-responders at 6 and 26 weeks had significantly lower baseline levels of N-acetyl aspartate (NAA) (both *p* = 0.01) and choline (*p*_6 weeks_ = 0.02; *p*_26 weeks_ = 0.006). In the larger 3.0×3.0×3.0 cm ACC MEGAPRESS voxel, baseline glx levels were lower in both responders (*p* = 0.006) and non-responders (*p* = 0.009) at 6 weeks and in responders at 26 weeks (*p* = 0.03) compared with HCs.

### Baseline glutamate and GABA levels and prediction of non-response

#### Predictive value of glutamate levels in the left thalamus

Higher baseline glutamate/Cr levels were significantly associated with the likelihood of being a non-responder at both 6 weeks [*p*_6 weeks_ = 0.031, *b* = 5.37, s.e. = 2.50, Wald χ^2^ = 4.36, OR = 2.2 (0.02–287) per 0.01 unit increase in glutamate/Cr, sensitivity = 33%], and 26 weeks [*p*_26 weeks_ = 0.026, *b* = 6.38, s.e. = 2.86, Wald χ^2^ = 4.98, OR = 5.9 (0.02–1594) per 0.01 unit increase in glutamate/Cr, sensitivity = 33%] compared with reference levels in HCs. The association remained significant after adjusting for age (*p*_6 weeks_ = 0.027; *p*_26 weeks_ = 0.021) and sex (*p*_6 weeks_ = 0.028; *p*_26 weeks_ = 0.026).

For glutamate levels in IU, a similar result was found for non-responders at 26 weeks, but not at 6 weeks.

#### Predictive value of glutamate levels in ACC

Baseline glutamate/Cr levels were not associated with the likelihood of being a non-responder (*p*_6weeks_ = 0.11; *p*_26 weeks_ = 0.39), and adjusting for potential confounders did not affect the results (*p*_6 weeks_ = 0.07−0.17; *p*_26 weeks_ = 0.17–0.46).

Similar results were seen for glutamate levels in IU.

#### Predictive value of GABA levels in ACC

Lower baseline GABA/Cr levels were significantly associated with the likelihood of being a non-responder at 6 weeks [*p* = 0.046, *b* = −65.4, s.e. = 32.8, Wald χ^2^ = 3.97, OR = 2.5×10^26^ (0.03–2.4×10^54^) per 0.01 unit decrease in GABA/Cr, sensitivity = 36%], and 26 weeks [*p* = 0.032, *b* = −81.6, s.e. = 38.1, Wald χ^2^ = 4.6, OR = 2.7×10^33^ (11.3–7.6×10^65^) per 0.01 unit decrease in GABA/Cr, sensitivity = 25%] compared with reference levels in HCs. The association remained significant after adjusting for age (*p*_6 weeks_ = 0.047; *p*_26 weeks_ = 0.030) and sex (*p*_6 weeks_ = 0.047; *p*_26 weeks_ = 0.019).

For GABA levels in IU, similar results were not found.

The accuracy of the prediction was slightly improved when both glutamate/Cr in the thalamus and GABA/Cr in ACC were included in the same model (percentage of correct identified non-responders at 6 weeks: 45.5%; and at 26 weeks: 37.5%).

### Baseline glutamate and GABA levels: associations with a symptom reduction of PANSS positive and total

Baseline levels of glutamate/Cr in the left thalamus were significantly negatively associated with the percent change in positive symptoms after 26 weeks (*N* = 24, R = −0.52, *p* = 0.01). The other correlations were insignificant.

## Discussion

To the best of our knowledge, this is the first longitudinal study of initially antipsychotic-naïve patients with first-episode psychosis investigating whether baseline glutamate and GABA levels are associated with treatment response compared with reference levels in HCs. The main results are that higher baseline levels of glutamate in the left thalamus and lower GABA levels in ACC are associated with poor treatment response at 6 and 26 weeks. The data did not, however, confirm our hypothesis of increased baseline glutamate levels in ACC of non-responders.

The finding that high thalamic glutamate levels and low ACC GABA levels at illness onset are associated with non-response at both 6 and 26 weeks compared to reference levels in HCs is clinically important. A normative reference interval of glutamate and GABA levels from HCs can be useful in guiding individual treatment choice in first-episode patients at illness onset, if the current findings are replicated. This could spare non-responders from the trial-and-error treatment approach with multiple dopaminergic antagonists, which they are currently subjected to. Interestingly, high thalamic glutamate levels and low GABA levels in ACC significantly predicted non-responders when compared with levels in HCs. However, the sensitivity was only 25–36%, but it might be enhanced if baseline glutamate and GABA levels are combined with other factors associated with non-response, such as striatal dopamine levels (Wulff *et al*., 2015, 2019), reward disturbances (Nielsen *et al*., 2012), cognitive measures (Green, 2006), and structural brain changes (Cahn *et al*., 2006) in future studies.

Our findings support that abnormal glutamate and GABA levels in initially antipsychotic-naïve patients are associated with non-response after first antipsychotic treatment as also reported in two recent studies, but the direction of abnormalities and regions affected differ (de la Fuente-Sandoval *et al*., 2017; Egerton *et al*., 2018). Egerton *et al*. found significantly higher glutamate levels at baseline in ACC, but not in the left thalamus of minimally-treated non-responder patients after 4 weeks treatment with amisulpride (Egerton *et al*., 2018), and de la Fuente-Sandoval *et al*. reported higher GABA levels in medial frontal cortex of non-responding antipsychotic-naïve patients after 4 weeks of treatment with risperidone (de la Fuente-Sandoval *et al*., 2017). These variations may be explained by methodical differences. Most importantly, we used matched HCs as a reference instead of responders. Second, we observed a decrease in thalamic glutamate levels after treatment was initiated and therefore speculate if the prior exposure to antipsychotic treatment had reduced thalamic glutamate levels in the study of minimally-treated patients (Egerton *et al*., 2018). Third, the voxel placement varied between studies. We placed our voxel in the area corresponding to the dorsal ACC, whereas the two other studies placed it in the pregenual ACC. Previous studies have reported a different distribution of glutamatergic and GABAergic receptors in these two subregions (Palomero-Gallagher *et al*., 2009), and higher glutamate and GABA levels in the pregenual ACC compared to the dorsal ACC in HCs (Dou *et al*., 2013). Interestingly, we also observed lower baseline glx in both responders and non-responders after 6 weeks in the larger and more dorsal MEGAPRESS voxel, but not in the PRESS voxel. It therefore seems likely that glutamatergic and GABAergic disturbances in ACC are region-specific in patients. Moreover, differences in factors that influence glutamate and GABA levels, including age (Marsman *et al*., 2013; Rowland *et al*., 2016), sex (O'Gorman *et al*., 2011), cigarette smoking (Akkus *et al*., 2013), and possibly also ethnicity and substance abuse, may contribute to the heterogeneous findings. Last, clozapine treatment in chronic patients might enhance glutamate levels as indicated by preclinical studies (Melone *et al*., 2001; Tanahashi *et al*., 2012) and by a recent cross-sectional study that found increased glx in dACC of non-responders to clozapine (Iwata *et al*., 2019).

Our second aim was to investigate the effect of 6 weeks of aripiprazole monotherapy on glutamate and GABA levels in initially antipsychotic-naïve patients. Aripiprazole has been shown to reduce glutamate release through activation of the dopamine D_2_ and 5-HT_1A_ receptors (Yang and Wang, 2008). Moreover, in preclinical models, where glutamate has been increased by the administration of a non-competitive NMDAR antagonist in the thalamus, aripiprazole appears to inhibit the cortical release of glutamate (Fukuyama *et al*., 2018). We found increased glutamate levels in the left thalamus at baseline and decreased ACC levels of GABA and glutamate at all assessments in the whole group of patients. Both aripiprazole monotherapy during 6 weeks and naturalistic antipsychotic treatment during 26 weeks decreased thalamic glutamate levels, but did not affect glutamate and GABA levels in ACC. This suggests that both aripiprazole and other antipsychotic compounds reduce glutamate levels in the thalamus, which is in line with the two existing previous studies of initially antipsychotic-naïve patients (Theberge *et al*., 2007; Aoyama *et al*., 2011). Future studies should investigate if a reduction of glutamate levels can prevent further loss of brain tissue in the thalamus. This is clinically relevant since a recent meta-analysis found reduced NAA in antipsychotic-naïve/free patients (Iwata *et al*., 2018) suggesting reduced neuronal viability and density from illness onset. In ACC, levels of GABA and glutamate were lower in the patients at all examinations and not affected by monotherapy with aripiprazole. The few existing longitudinal studies of first-episode patients have reported equivocal results of the effect of treatment on glutamate and GABA levels in ACC. Three studies have reported no effect on glutamate levels after treatment with different antipsychotic compounds (Theberge *et al*., 2007; Bustillo *et al*., 2010; Aoyama *et al*., 2011), and two studies have reported reductions in glutamate (Egerton *et al*., 2018) and GABA levels (de la Fuente-Sandoval *et al*., 2017) after monotherapy with amisulpride and risperidone, respectively. It is possible that only certain antipsychotic compounds reduce levels of glutamate and GABA in ACC as indicated by preclinical studies (Bustillo *et al*., 2006; Yang and Wang, 2008; Xu *et al*., 2015) and that reduction of glutamatergic metabolites mainly occur when levels are abnormally increased.

Strengths of the present study are a large sample of strictly antipsychotic-naïve patients with first-episode psychosis, measures of both glutamate and GABA levels, and follow-up examinations at both 6 and 26 weeks. Some limitations should, however, be considered. First, we used response criteria originally developed for remission in the chronic phase of schizophrenia (Andreasen *et al*., 2005). Second, the association between cellular mechanisms and 1H-MRS measures of whole tissue glutamate and GABA remains to be elucidated, and we cannot determine whether the MRS measures reflect glutamatergic abnormalities in synaptic transmission or metabolism (Rothman *et al*., 2011). Third, our GABA findings reflect GABA + macromolecules, with the latter being a potential confounder (Rothman *et al*., 1993). Last, we used glutamate/Cr as the main outcome. In 1H-MRS, total creatine (PCr + Cr) or water-scaled values corrected for CSF content in the voxel are commonly used as references, and at present, there is no consensus regarding the superiority of either method. The PCr + Cr peak at 3.03 ppm is considered stable despite brain pathologies because Cr plus PCr are in equilibrium (Saunders *et al*., 1999). Despite this, we observed that PCr + Cr in the thalamus was significantly lower at baseline in non-responders at 6 weeks compared with HCs. Therefore, we cannot rule out that baseline differences in PCr + Cr between non-responders at 6 weeks and HCs influenced thalamic levels of glutamate/Cr at baseline. However, no differences in PCr + Cr in the thalamus at baseline was observed in non-responders at 26 weeks compared with HCs, neither any baseline differences in PCr + Cr in the MEGAPRESS voxel measuring GABA, why it appears unlikely that the present findings can be explained by alterations in total creatine.

In conclusion, our findings suggest that increased glutamatergic neurotransmission in the thalamus and decreased GABAergic neurotransmission in ACC are part of the pathophysiology of first-episode psychosis and that aberrations in these neurotransmitters are driven by patients, who will not respond to antipsychotic treatment. Furthermore, monotherapy with aripiprazole and naturalistic antipsychotic treatment reduce glutamate levels in the thalamus, whereas glutamate and GABA levels in ACC are unaffected. If replicated, normative reference intervals for glutamate and GABA levels may aid estimation of clinical prognosis in patients with first-episode psychosis.

## Supplementary material

For supplementary material accompanying this paper visit https://doi.org/10.1017/S0033291719002277. click here to view supplementary material
