# Supplementary material for: Treatment response after 6 and 26 weeks is related to baseline glutamate and GABA levels in antipsychotic-naïve patients with psychosis
Source: Psychol Med. 2019 Sep 16;50(13):2182–93. doi: 10.1017/S0033291719002277 (PMC7557159; doi:10.1017/S0033291719002277)
Supplement: Supplementary file 1 [file S0033291719002277sup001.docx]

**Supplementary Material**

**Supplementary Methods**

**Participants and design**

The present study is part of an ongoing prospective, multimodal longitudinal study denoted the Pan European Collaboration on Antipsychotic Naïve Schizophrenia II (PECANSII), in which initially antipsychotic-naïve patients and matched healthy controls (HCs) are examined at baseline and after 6, 26, and 104 weeks of treatment. Data on 104 weeks are not part of the current work. In addition to magnetic resonance spectroscopy (MRS), participants undergo magnetic resonance imaging (MRI) including structural and functional MRI sequences, 3,4-dihydroxy-6-[18F] fluor-L-phenylalanine (^18^F-DOPA) positron emission tomography (PET) (only at baseline and 6 weeks; PET was initiated about 1.5 year after the other examinations), electrophysiological examinations, and neurocognitive testing. Apart from PET at 6 weeks, HCs undergo similar examinations as patients but are not treated with antipsychotic medication. Baseline data are also part of a recently published paper describing patterns of cortical structures and cognition at baseline (Jessen *et al.*, 2018).

The diagnostic SCAN interview was performed by trained interviewers (two doctors or a doctor and research nurse), and the diagnosis was re-assessed by a psychiatrist. Patients were only included if agreement on a diagnosis of schizophrenia, schizoaffective disorder, or non-organic psychosis was obtained and there was a need for antipsychotic treatment. Further exclusion criteria for patients were treatment with an antidepressant within the last 30 days and being involuntary admitted. HCs were excluded if they had a lifetime psychiatric illness, first-degree relatives with a psychiatric diagnosis, or fulfilled the criteria for ultra-high-risk psychosis as evaluated with the Comprehensive Assessment of At-Risk Mental States (Yung *et al.*, 1998). Exclusion criteria for all participants were a history of a head injury with unconsciousness >5 min, severe medical condition, and contraindication to MRI scans.

**Clinical assessments**

Treatment outcomes were evaluated with the Andreasen criteria, in which remission is defined as a score of mild or less (< 3) on the following 8 PANSS scores: delusion (P1), conceptual disorganisation (P2), hallucinatory behaviour (P3), blunted affect (N1), social withdrawal (N4), lack of spontaneity (N6), mannerisms/posturing (G5), and unusual though content (G9) (Andreasen *et al.*, 2005).

**MRI**

MRI was performed on a 3.0 Tesla scanner (Achieva; Philips Healthcare, Eindhoven, The Netherlands) using a 32-channel head coil (Invivo, Orlando, Florida, USA). Participants were instructed not to move their head or body during the scans. A high-resolution, three-dimensional, T1-weighted structural image of the brain (TR 10 ms, TE 4.6 ms, flip angle = 8°, voxel size = 0.79 × 0.79 × 0.80mm) was acquired for anatomic reference, spectroscopic voxel placement, and tissue segmentation.

**MRI scanner upgrade**

After study initiation, a technical upgrade was performed on the MRI scanner for 8 months. Before the upgrade, 10 patients and 4 HCs were scanned at baseline, 8 patients and 4 HCs were scanned at 6 weeks, and 5 patients were scanned at 26 weeks. Glutamate/Cr levels in anterior cingulate cortex (ACC) of 8 healthy subjects scanned before and after the scanner upgrade did not differ (T[7]=1.26, p=0.25).

**MRS voxel placements and coefficients of variation**

*PRESS*: A 2.0x2.0x2.0cm voxel was placed in the ACC by first drawing a line through the extremities of the corpus callosum and then placing the voxel corner where the line crossed the genu of the corpus callosum, and, finally, the voxel was aligned with the corpus callosum as shown in Figure S1B. To avoid signal interference, saturation bands were used to cover lipids in the sculp and water in the circulus willisii. A 2.0x1.5x2.0cm voxel was placed in left thalamus so that a minimum amount of cerebrospinal fluid (CSF) was included in the voxel, as shown in Figure S1A. The acquisition time for both PRESS sequences was 14 minutes. Representative LCModel (Provencher, 1993) spectra from left thalamus and ACC, respectively, are shown in Figures S1D and E.

*MEGAPRESS*: A 3.0x3.0x3.0cm voxel in ACC was placed as shown in Figure S1C, following the same procedure as described above. The acquisition time for the MEGAPRESS sequence was 11 minutes. Figure S1F shows a representative spectra from Gannet (Edden *et al.*, 2014).

**Figure S1.** **Voxel locations and representative spectra.**


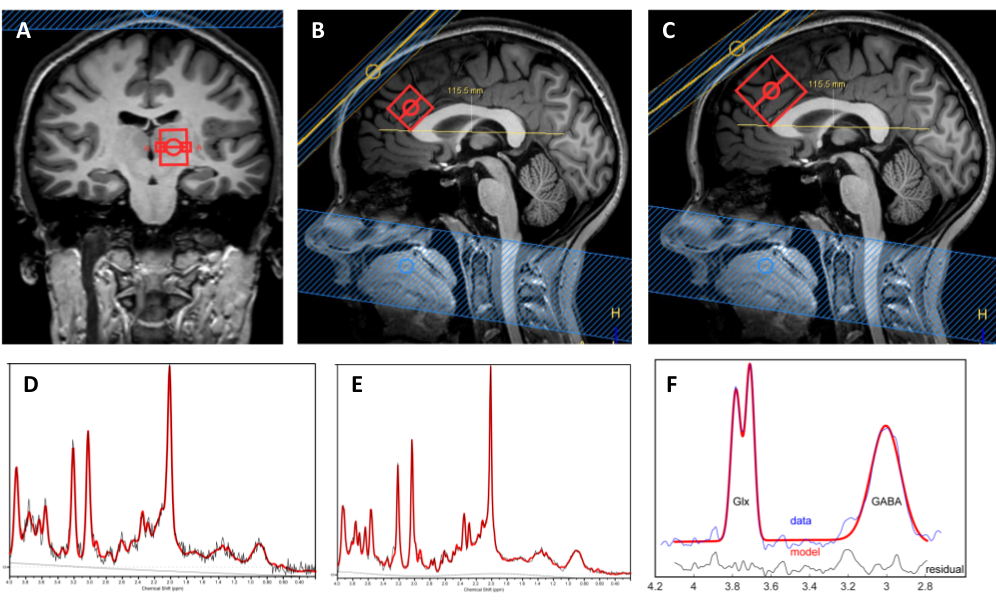


PRESS acquisitions: The location of the left thalamus voxel is shown in A and the anterior cingulate cortex (ACC) voxel in B, along with representative spectra from analyses in LCModel in D for the left thalamus, and in E for the ACC. MEGAPRESS: The location of the ACC voxel is shown in C along with representative spectra from the analysis in Gannet in F.
Abbreviations: Glx: Glutamate + glutamine; GABA: Gamma-aminobutyric acid.

The coefficient of variation (CV%) calculated from the repeated MRS scans of HCs were for glutamate/Cr levels in ACC 5.2% after 6 and 26 weeks, for glutamate/Cr levels in left thalamus 8.1% after 6 weeks and 8.8% after 26 weeks, and for GABA/Cr levels in ACC 7.3% after 6 weeks and 10.7% after 26 weeks.

**Segmentation and calculation of MRS voxel tissue content**

The voxel percentage of grey and white matter was extracted by segmenting the T1-weighted structural image using SPM 8 (<http://www.fil.ion.ucl.ac.uk/spm/software/spm8/>) running under MATLAB® (The

MathWorks Inc., Massachusetts, USA) and combining the segmentation with the MRS voxel masks from the PRESS and MEGAPRESS acquisitions. This was used to correct in vivo water-scaled values of metabolites reported by LCModel and Gannet for partial volume CSF contamination and to calculate metabolite concentrations in IU corrected for partial volume CSF contamination using the following equation (Stone *et al.*, 2012):

M_IU_ = M*(wm+gm+1.55*CSF)/(wm+gm),

where ‘M’ is in vivo water-scaled values of metabolites reported by LCModel and Gannet; ‘wm’ and ‘gm’ are fractions of white and grey matter in the spectroscopic voxel; and ‘CSF’ is the CSF content in the spectroscopic voxel calculated as 1-(wm+gm). For a full description, see (Stone *et al.*, 2012).

**Supplementary Results**

**Participant characteristics**

At 6 weeks, 12 patients had dropped out due to akathisia (2), other side effects (2), loss of contact (1), insufficient antipsychotic effect of aripiprazole (2), mistakenly treated with another antipsychotic compound or antidepressants (3), or because the patient wanted to terminate participation in the study (2). One HC also wanted to terminate participation in the study. At 26 weeks, 11 patients had dropped out due to loss of contact (3) or because the patients wanted to terminate participation in the study (8). Two HCs also dropped out, one due to loss of contact (1) and one wanted to terminate participation in the study (1).

Baseline glutamate/Cr and GABA/Cr levels did not differ between drop-out patients and study completers in ACC (ACC glutamate: p_6weeks_=0.73, p_26weeks_=0.40; ACC GABA: p_6weeks_=0.50, p_26weeks_=0.27) or in left thalamus for the 6 weeks examinations (p=0.73), but baseline glutamate/Cr levels were significantly lower in left thalamus of patients who dropped out of the 26 weeks examinations (T[36]=2.29, p=0.03).

**Proton magnetic resonance spectroscopy (1H-MRS) quality**

The spectral quality of the 1H-MRS data and the fit of individual metabolites were judged good as described in detail below. Signal-to-noise ratios, full-width half-maximum, and CRLB values are summarised for baseline data in Tables S2 and S3, for 6 weeks data in Table S4 and S5, and for 26 weeks data in Tables S6 and S7.

*1H-MRS baseline data:*

At baseline, 44 patients and 36 HCs were recruited. Of these, 5 patients did not get a MRI scan due to a technical upgrade of the scanner.

**Thalamus_PRESS_**: Three spectra were excluded from left thalamus by visual inspection (2 HCs and 1 patient) and 1HC did not get the acquisition due to technical problems with the sequence leaving a total of 38 patients and 33 HCs spectra. For individual metabolites, exclusion due to Cramér-Rao lower bound (CRLB) >20% was done for 1 glutamate (1 HC) and 62 glutamine (which is why glutamine is not reported at all for the left thalamus) data, whereas no CRLB values >20% were observed for glutamate + glutamine (glx), N-acetyl aspartate (NAA), Cr+PCr (total creatine used as reference by LCModel), myo-inositol, or choline. Table S2 summarizes the spectral quality data.

**ACC_PRESS_**: Two spectra from the ACC_PRESS_ (2 patients) were excluded by visual inspection, leaving a total of 37 patients and 36 HCs spectra. Exclusion due to CRLB >20% was done for 14 glutamine data (9 HCs and 5 patients), whereas no CRLB values >20% was observed for glx, NAA, Cr+PCr, myo-inositol, and choline. Table S2 summarises the spectral quality data.

**ACC_MEGAPRESS_**: Six spectra from the ACC_MEGAPRESS_ (2 HCs and 4 patients) were excluded by visual inspection, and in one spectrum the water was not quantifiable (1 patient) and therefore only the creatine scaled values, but not the water scaled values could be used. Nine participants did not get the MEGAPRESS scan (2 HCs and 6 patients) due to technical problems with the sequence and discomfort in the scanner (1 patient), leaving a total of 28 patients with creatine scaled values and 27 patients with water scaled values and 32 HCs spectra. There were no fit-error values >15% for GABA or glx. Table S3 summarises the spectral quality data.

*1H-MRS at 6 weeks:*

At 6 weeks, 32 patients and 35 HCs completed the visit. Of these, 1 patient did not get a MRI scan due to a technical upgrade of the scanner.

**Thalamus_PRESS_**: Six spectra were excluded by visual inspection (2 HCs and 4 patients), and 5 participants did not get the acquisition due to technical problems with the sequence (2 HCs) and discomfort in the scanner (3 patients), leaving a total of 24 patients and 31 HCs spectra. Exclusion due to CRLB >20% was done for 65 glutamine data (which is why glutamine is not reported at all for the left thalamus), but no CRLB values >20% were found for glutamate, glx, NAA, Cr+PCr (total creatine used as reference by LCModel), myo-inositol, or choline. Table S4 summarises the spectral quality data.

**ACC_PRESS_**: Three spectra were excluded by visual inspection (1 HCs and 2 patients), and 4 participants did not get the acquisition due to technical problems with the sequence (2 HCs) and discomfort in the scanner (2 patients), leaving a total of 27 patients and 32 HCs spectra. Exclusion due to CRLB >20% was done for 8 glutamine data (3 HCs and 5 patients), but no CRLB values >20% were found for glutamate, glx, NAA, Cr+PCr (total creatine used as reference by LCModel), myo-inositol, or choline. Table S4 summarises the spectral quality data.

**ACC_MEGAPRESS_**: One spectrum was excluded by visual inspection (1 patient), and 12 participants did not get the acquisition due to technical problems with the sequence (2 HCs and 6 patients), data that was mistakenly not transferred (1 HCs), and discomfort in the scanner (4 patients). There were no fit-error values >15% for GABA or glx. Table S5 summarises the spectral quality data.

*1H-MRS at 26 weeks:*

At 26 weeks, 30 patients and 32 HCs completed the visit. Of these, 4 patients and 1 HC did not get a MRI scan due to discomfort in the scanner initially after placement (3 patients), loss of contact after PANSS assessment (1 patient), and due to a technical upgrade of the scanner (1HC).

**Thalamus_PRESS_**: Four spectra were excluded by visual inspection from the left thalamus (3 HCs and 1 patient), and 2HC did not get the thalamus_PRESS_ scan due to technical problems with the sequence, leaving a total of 25 patients and 26 HCs spectra. Exclusion due to CRLB >20% was done for 46 glutamine data (which is why glutamine is not reported at all for left thalamus), but no CRLB values > 20% were found for glutamate, glx, NAA, Cr+PCr, myo-inositol, or choline. Table S6 summarises the spectral quality data.

**ACC_PRESS_**: No spectra were excluded after visual inspection, leaving a total of 26 patients and 31 HCs spectra. Exclusion due to CRLB >20% was done for 7 glutamine data (2 HCs and 5 patients), but no CRLB values >20% were found for glutamate, glx, NAA, Cr+PCr, myo-inositol, or choline. Table S6 summarises the spectral quality data.

**ACC_MEGAPRESS_**: Three spectra were excluded after visual inspection (1 patient, 2 HCs) and 1 patient did not get the acquisition due to discomfort in the scanner, leaving a total of 24 patients and 29 HCs spectra. There were no fit-error values >15% for GABA or glx. Table S7 summarises the spectral quality data.

*Segmentation:*

The segmentation of grey and white matter could not be done for 1 HC at the 6 weeks examination.

**The effect of treatment on glutamate and GABA levels in IU**

*Left thalamus*

*Glutamate IU*: There was trend toward a group*time interaction (F[2,60.6]=2.47, p=0.09) due to a trend for increased glutamate IU in patients at baseline (T[68.7]=1.70, p=0.09, estimate=0.34 [CI:-0.06 – 0.74]), whereas there were no differences between groups at 6 weeks (p=0.26) and 26 weeks (p=0.19). A similar trend for an interaction was also observed after adjusting for age (p=0.09), sex (p=0.08), smoking status (p=0.09), cannabis use (p=0.09), and scanner upgrade (p=0.12).

*ACC*

*Glutamate*: The group*time interaction and main effects of group and time were both insignificant (p=0.52-0.91). The main effect was also insignificant when removing the group*time interaction and adjusting for age, sex, smoking status, cannabis use, and scanner upgrade (all p>0.05).

*GABA*: The group*time interaction was insignificant (p=0.61). There was a borderline significant main effect of group due to lower GABA in patients (F[1,73.3]=3.63, p=0.06), also after removing the group*time interaction (p=0.05). The main effect of group was significant when adjusting for sex (p=0.04), but only trend-level significant when adjusting for age (p=0.05), smoking status (p=0.07), cannabis use (p=0.06), scanner upgrade (p=0.06), and the exclusion of subjects who regularly used benzodiazepines occasionally or regularly (p=0.09).

**Baseline glutamate and GABA levels in IU in non-responders and responders compared with healthy controls**

*Baseline glutamate levels in IU in left thalamus*

Baseline glutamate levels in IU in thalamus were significantly higher in patients not responding to treatment after 26 weeks compared with HCs, although the overall model only was trend-level significant, whereas there was no difference between HCs and responding patients (overall model: F(2,49=2.54), p=0.09, post hoc test: p_Non-responder_=0.04, p_Responder_=0.20; overall model after adjustment for age and sex: p=0.08, post hoc test: p_Non-responder_=0.03, p_Responder_=0.28). There were no significant differences in baseline IU values neither between patients who were non-responders nor responders after 6 weeks and HCs (overall model: p=0.38; after adjustment for age and sex: p=0.58).

*Baseline glutamate levels in IU in ACC*

For glutamate levels in IU in ACC, there were no baseline differences between patients not responding at 6 weeks or 26 weeks and HCs, nor between responders at 6 weeks or 26 weeks and HCs (Overall models: p-values with and without adjustment for age and sex: 0.19-0.85).

*Baseline GABA levels in IU in ACC*

For GABA levels in IU in ACC, there were no baseline differences between patients not responding at 6 weeks or 26 weeks and HCs, nor between responders at 6 weeks or 26 weeks and HCs (Overall models: p-values with and without adjustment for age and sex: 0.06-0.58).

**Baseline glutamate and GABA levels in IU and prediction of non-response**

*Predictive value of glutamate levels in IU in left thalamus*

Higher glutamate levels in IU were borderline significantly associated with the likelihood of being a non-responder at 26 weeks (p=0.05, b=0.92, SE=0.47, Wald χ^2^=3.82) and significantly when adjusting for age and sex (p=0.034-0.049), but the association was not significant for non-responders at 6 weeks (p=0.28; after adjustment for age and sex: p=0.20-0.32).

*Predictive value of glutamate levels in IU in ACC*

Glutamate levels in IU were not significantly associated with the likelihood of being a non-responder at 6 weeks (p=0.36) or at 26 weeks (p=0.84) and adjustment for age and sex did not change the result (p_6 weeks_=0.29 -0.55; p_26 weeks_=0.51-0.86).

*Predictive value of GABA levels in IU in ACC*

GABA levels in IU were not significantly associated with the likelihood of being a non-responder at 6 weeks (p=0.36) or at 26 weeks (p=0.24) and adjustment for age and sex did not change the result (p_6 weeks_=0.34 -0.43; p_26 weeks_=0.18-0.37).

**References**

**Andreasen, N. C., Carpenter, W. T., Jr., Kane, J. M., Lasser, R. A., Marder, S. R. & Weinberger, D. R.** (2005). Remission in schizophrenia: proposed criteria and rationale for consensus. American Journal of Psychiatry 162, 441-9.

**Edden, R. A., Puts, N. A., Harris, A. D., Barker, P. B. & Evans, C. J.** (2014). Gannet: A batch-processing tool for the quantitative analysis of gamma-aminobutyric acid-edited MR spectroscopy spectra. Journal of Magnetic Resonance Imaging 40, 1445-52.

**Jessen, K., Mandl, R. C. W., Fagerlund, B., Bojesen, K. B., Raghava, J. M., Obaid, H. G., Jensen, M. B., Johansen, L. B., Nielsen, M. O., Pantelis, C., Rostrup, E., Glenthoj, B. Y. & Ebdrup, B. H.** (2018). Patterns of Cortical Structures and Cognition in Antipsychotic-Naive Patients With First-Episode Schizophrenia: A Partial Least Squares Correlation Analysis. Biological Psychiatry: Cognitive Neuroscience and Neuroimaging.

**Provencher, S. W.** (1993). Estimation of metabolite concentrations from localized in vivo proton NMR spectra. Magnetic Resonance in Medicine 30, 672-9.

**Stone, J. M., Dietrich, C., Edden, R., Mehta, M. A., De Simoni, S., Reed, L. J., Krystal, J. H., Nutt, D. & Barker, G. J.** (2012). Ketamine effects on brain GABA and glutamate levels with 1H-MRS: relationship to ketamine-induced psychopathology. Molecular Psychiatry 17, 664-5.

**Yung, A. R., Phillips, L. J., McGorry, P. D., McFarlane, C. A., Francey, S., Harrigan, S., Patton, G. C. & Jackson, H. J.** (1998). Prediction of psychosis. A step towards indicated prevention of schizophrenia. British Journal of Psychiatry Supplement 172, 14-20.

**Table S1. Diagnoses and side effects**

|  | **Baseline** | **6 weeks** | **26 weeks** | **Statistics** (Baseline vs 6 and 26 weeks) |
| --- | --- | --- | --- | --- |
| **Diagnosis, n**  Schizophrenia  Undifferentiated  Paranoid  Disorganised  Simplex  Schizoaffective, depressive  Non-organic psychosis | 11  23  4  1  2  3 |  |  |  |
| **UKU side effects, mean ± SD:** |  |  |  |  |
| **Total score** | 29.1 ± 15.1 | 19.5 ± 7.8******* | 17.1 ± 9.6******* | F(2,27.5)=12.74, p<0.0001**^A^** |
| **Psychiatric** | 14.4 ± 5.7 | 9.7 ± 3.8******* | 8.6 ± 5.3******* | F(2,25.4)=11.46, p=0.0003**^A^** |
| **Neurological** | 5.6 ± 4.2 | 3.8 ± 2.8***** | 2.8 ± 2.3******* | F(2,31.7)=7.98, p=0.002**^A^** |
| **Autonomic** | 4.3 ± 4.0 | 3.3 ± 2.3 | 2.4 ± 2.4***** | F(2,27.8)=3.67, p=0.04**^A^** |
| **Sexual** | 1.1 ± 2.0 | 0.6 ± 1.0 | 1.0 ± 1.6 | F(2,28.5)=2.00, p=0.15**^A^** |
| **Other** | 4.0 ± 3.5 | 2.3 ± 1.7****** | 2.5 ± 2.0****** | F(2,30.0)=5.30, p=0.01**^A^** |

Abbreviations: UKU: Task Force for Clinical Investigations (*Udvalget for Kliniske Undersøgelser*). A: Main effect of time; *: p<0.05; **: p<0.01; ***: p<0.001.

**Table S2. Spectral quality for PRESS acquisitions in ACC and left thalamus at baseline**

|  | **ACC** | |  | **Left thalamus** | |  |
| --- | --- | --- | --- | --- | --- | --- |
|  | **FEP** | **HCs** | **Statistics** | **FEP** | **HCs** | **Statistics** |
|  | **Mean ± SD, n^A^** | **Mean ± SD, n^A^** |  | **Mean ± SD, n^A^** | **Mean ± SD, n^A^** |  |
| **FWHM** | 0.040 ± 0.058, n=37 | 0.026 ± 0.007, n=36 | P=0.14 | 0.048 ± 0.007, n=38 | 0.047 ± 0.007, n=33 | P=0.85 |
| **Signal to noise ratio** | 31.3 ± 3.3, n=37 | 31.7 ± 3.2, n=36 | P=0.61 | 15.6 ± 3.6, n=38 | 16.1 ± 3.3, n=33 | P=0.55 |
| **CRLB (%) glutamate** | 5.2 ± 0.7, n=37 | 5.1 ± 0.5, n=36 | P=0.72 | 9.6 ± 2.2, n=38 | 10.1 ± 2.1, n=32 | P=0.38 |
| **CRLB (%) Glx** | 4.9 ± 0.9, n=37 | 5.0 ± 0.6, n=36 | P=0.76 | 8.3 ± 1.5, n=38 | 8.7 ± 1.7, n=33 | P=0.38 |
| **CRLB (%) glutamine** | 16.1 ± 2.4, n=32 | 16.1 ± 2.2, n=27 | P=0.97 | - | - | - |
| **CRLB (%) NAA** | 3.1 ± 0.4, n=37 | 3.1 ± 0.3, n=36 | P=0.78 | 5.4 ± 1.6, n=38 | 5.1 ± 0.9, n=33 | P=0.32 |
| **CRLB (%) PCr+Cr** | 2.9 ± 0.2, n=37 | 3.0 ± 0.0, n=36 | P=0.16 | 4.1 ± 1.0, n=38 | 3.9 ± 0.8, n=33 | P=0.28 |
| **CRLB (%) myo-inositol** | 4.1 ± 0.4, n=37 | 4.1 ± 0.5, n=36 | P=0.97 | 8.6 ± 2.3, n=38 | 8.2 ± 1.8, n=33 | P=0.44 |
| **CRLB (%) choline** | 3.2 ± 0.4, n=37 | 3.1 ± 0.2, n=36 | P=0.15 | 4.8 ± 1.3, n=38 | 4.5 ± 0.8, n=33 | P=0.24 |

Abbreviations: ACC: Anterior cingulate cortex; FEP: First episode psychosis; HCs: Healthy controls; FWHM: Full-width half-maximum; CRLB: Cramér-Rao lower bound; Glx: Glutamate+glutamine; NAA: N-acetyl aspartate; PCr+Cr: Total creatine (creatine+phosphocreatine). A: n states the number of analyzed spectra.

**Table S3. Spectral quality for MEGAPRESS acquisitions in ACC at baseline**

|  | **ACC** | |  |
| --- | --- | --- | --- |
|  | **FEP** | **HCs** | **Statistics** |
|  | **Mean ± SD, n^A^** | **Mean ± SD, n^A^** |  |
| **FWHM** | 19.7 ± 2.1, n=28 | 20.1 ± 1.4, n=32 | P=0.49 |
| **Signal to noise ratio** | 20.7 ± 4.5, n=28 | 21.4 ± 5.2, n=32 | P=0.58 |
| **Fit error (%) GABA** | 6.5 ± 1.3, n=28 | 6.4 ± 1.5, n=32 | P=0.87 |
| **Fit error (%) Glx** | 3.1 ± 0.9, n=28 | 2.9 ± 0.7, n=32 | P=0.24 |

Abbreviations: ACC: Anterior cingulate cortex; FEP: First episode psychosis; HCs: Healthy controls; FWHM: Full-width half-maximum; GABA: Gamma-aminobutyric acid; Glx: Glutamate+glutamine. A: n states the number of analyzed spectra.

**Table S4. Spectral quality for PRESS acquisitions in ACC and left thalamus at 6 weeks**

|  | **ACC** | |  | **Left thalamus** | |  |
| --- | --- | --- | --- | --- | --- | --- |
|  | **FEP** | **HCs** | **Statistics** | **FEP** | **HCs** | **Statistics** |
|  | **Mean ± SD, n^A^** | **Mean ± SD, n^A^** |  | **Mean ± SD, n^A^** | **Mean ± SD, n^A^** |  |
| **FWHM** | 0.030 ± 0.011, n=27 | 0.027 ± 0.004, n=32 | P=0.16 | 0.048 ± 0.008, n=24 | 0.046 ± 0.005, n=31 | P=0.29 |
| **Signal to noise ratio** | 30.3 ± 4.0, n=27 | 31.6 ± 2.9, n=32 | P=0.15 | 15.5 ± 3.4, n=24 | 16.0 ± 3.4, n=31 | P=0.65 |
| **CRLB (%) glutamate** | 5.5 ± 1.3, n=27 | 5.2 ± 0.4, n=32 | P=0.26 | 10.1 ± 1.4, n=24 | 10.3 ± 1.9, n=31 | P=0.71 |
| **CRLB (%) Glx** | 5.2 ± 1.5, n=27 | 5.0 ± 0.6, n=32 | P=0.42 | 8.7 ± 0.9, n=24 | 8.5 ± 1.3, n=31 | P=0.38 |
| **CRLB (%) glutamine** | 15.8 ± 2.3, n=22 | 16.7 ± 2.3, n=29 | P=0.18 | - | - | - |
| **CRLB (%) NAA** | 3.1 ± 0.4, n=27 | 3.1 ± 0.3, n=32 | P=0.53 | 5.4 ± 1.2, n=24 | 5.1 ± 0.8, n=31 | P=0.31 |
| **CRLB (%) PCr+Cr** | 3.0 ± 0.2, n=27 | 2.9 ± 0.2, n=32 | P=0.66 | 4.0 ± 0.8, n=24 | 4.0 ± 0.8, n=31 | P=0.96 |
| **CRLB (%) myo-inositol** | 4.3 ± 0.7, n=27 | 4.2 ± 0.5, n=32 | P=0.28 | 8.8 ± 2.1, n=24 | 8.7 ± 2.7, n=31 | P=0.85 |
| **CRLB (%) choline** | 3.3 ± 0.5, n=27 | 3.0 ± 0.2, n=32 | P=0.04 | 4.5 ± 0.8, n=24 | 4.5 ± 0.9, n=31 | P=0.83 |

Abbreviations: ACC: Anterior cingulate cortex; FEP: First episode psychosis; HCs: Healthy controls; FWHM: Full-width half-maximum; CRLB: Cramér-Rao lower bound; Glx: Glutamate+glutamine; NAA: N-acetyl aspartate; PCr+Cr: Total creatine (creatine+phosphocreatine). A: n states the number of analyzed spectra.

**Table S5. Spectral quality for MEGAPRESS acquisitions in ACC at 6 weeks**

|  | **ACC** | |  |
| --- | --- | --- | --- |
|  | **FEP** | **HCs** | **Statistics** |
|  | **Mean ± SD, n^A^** | **Mean ± SD, n^A^** |  |
| **FWHM** | 19.8 ± 1.5, n=20 | 19.9 ± 1.3, n=32 | P=0.33 |
| **Signal to noise ratio** | 21.2 ± 4.0, n=20 | 22.4 ± 4.4, n=32 | P=0.58 |
| **Fit error (%) GABA** | 6.4 ± 1.4, n=20 | 6.0 ± 1.4, n=32 | P=0.34 |
| **Fit error (%) Glx** | 2.8 ± 0.7, n=20 | 3.0 ± 0.9, n=32 | P=0.27 |

Abbreviations: ACC: Anterior cingulate cortex; FEP: First episode psychosis; HCs: Healthy controls; FWHM: Full-width half-maximum; GABA: Gamma-aminobutyric acid; Glx: Glutamate+glutamine. A: n states the number of analyzed spectra.

**Table S6. Spectral quality for PRESS acquisitions in ACC and left thalamus at 26 weeks**

|  | **ACC** | |  | **Left thalamus** | |  |
| --- | --- | --- | --- | --- | --- | --- |
|  | **FEP** | **HCs** | **Statistics** | **FEP** | **HCs** | **Statistics** |
|  | **Mean ± SD, n^A^** | **Mean ± SD, n^A^** |  | **Mean ± SD, n^A^** | **Mean ± SD, n^A^** |  |
| **FWHM** | 0.032 ± 0.009, n=26 | 0.026 ± 0.006, n=31 | P=0.004 | 0.046 ± 0.005, n=25 | 0.046 ± 0.006, n=26 | P=0.86 |
| **Signal to noise ratio** | 30.7 ± 3.4, n=26 | 31.7 ± 2.0, n=31 | P=0.16 | 16.0 ± 3.0, n=25 | 16.1 ± 2.9, n=26 | P=0.85 |
| **CRLB (%) glutamate** | 5.2 ± 0.5, n=26 | 5.1 ± 0.3, n=31 | P=0.25 | 9.9 ± 1.6, n=25 | 9.4 ± 1.1, n=26 | P=0.17 |
| **CRLB (%) Glx** | 5.2 ± 0.7, n=26 | 4.8 ± 0.5, n=31 | P=0.04 | 8.7 ± 1.6, n=25 | 8.2 ± 1.0, n=26 | P=0.14 |
| **CRLB (%) glutamine** | 16.3 ± 2.0, n=21 | 16.3 ± 2.3, n=29 | P=0.99 | - | - | - |
| **CRLB (%) NAA** | 3.0 ± 0.2, n=26 | 3.0 ± 0.0, n=31 | P=0.33 | 5.1 ± 0.9, n=25 | 5.0 ± 0.8, n=26 | P=0.62 |
| **CRLB (%) PCr+Cr** | 2.9 ± 0.3, n=26 | 3.0 ± 0.2, n=31 | P=0.48 | 3.9 ± 0.7, n=25 | 3.9 ± 0.6, n=26 | P=0.98 |
| **CRLB (%) myo-inositol** | 4.2 ± 0.7, n=26 | 4.0 ± 0.3, n=31 | P=0.13 | 8.3 ± 1.4, n=25 | 8.2 ± 2.1, n=26 | P=0.86 |
| **CRLB (%) choline** | 3.0 ± 0.5, n=26 | 3.0 ± 0.0, n=31 | P=1.00 | 4.5 ± 0.7, n=25 | 4.5 ± 0.7, n=26 | P=0.92 |

Abbreviations: ACC: Anterior cingulate cortex; FEP: First episode psychosis; HCs: Healthy controls; FWHM: Full-width half-maximum; CRLB: Cramér-Rao lower bound; Glx: Glutamate+glutamine; NAA: N-acetyl aspartate; PCr+Cr: Total creatine (creatine+phosphocreatine). A: n states the number of analyzed spectra.

**Table S7. Spectral quality for MEGAPRESS acquisitions in ACC at 26 weeks**

|  | **ACC** | |  |
| --- | --- | --- | --- |
|  | **FEP** | **HCs** | **Statistics** |
|  | **Mean ± SD, n^A^** | **Mean ± SD, n^A^** |  |
| **FWHM** | 20.0 ± 1.4, n=24 | 20.1 ± 2.0, n=29 | P = 0.72 |
| **Signal to noise ratio** | 21.5 ± 6.2, n=24 | 21.9 ± 3.7, n=29 | P = 0.76 |
| **Fit error (%) GABA** | 6.1 ± 1.4, n=24 | 6.2 ± 1.2, n=29 | P = 0.83 |
| **Fit error (%) Glx** | 3.5 ± 1.1, n=24 | 2.9 ± 0.7, n=29 | P = 0.03 |

Abbreviations: ACC: Anterior cingulate cortex; FEP: First episode psychosis; HCs: Healthy controls; FWHM: Full-width half-maximum; GABA: Gamma-aminobutyric acid; Glx: Glutamate+glutamine. A: n states the number of analyzed spectra.

**Table S8. Metabolites scaled to total creatine in ACC and left thalamus at all assessments in first-episode psychosis patients and healthy controls**

|  | **Baseline** | | **6 weeks** | | **26 weeks** | |
| --- | --- | --- | --- | --- | --- | --- |
|  | **FEP** | **HCs** | **FEP** | **HCs** | **FEP** | **HCs** |
| ***Thalamus*** | **Mean ± SD, n^A^** | **Mean ± SD, n^A^** | **Mean ± SD, n^A^** | **Mean ± SD, n^A^** | **Mean ± SD, n^A^** | **Mean ± SD, n^A^** |
| **Glutamate/Cr** | 1.29 ± 0.15, n=38 | 1.22 ± 0.15, n=32 | 1.20 ± 0.14, n=24 | 1.23 ± 0.14, n=31 | 1.21 ± 0.15, n=25 | 1.27 ± 0.12, n=26 |
| **Glx/Cr** | 1.81 ± 0.27, n=38 | 1.77 ± 0.22, n=33 | 1.70 ± 0.26, n=24 | 1.80 ± 0.22, n=31 | 1.74 ± 0.27, n=25 | 1.82 ± 0.21, n=26 |
| **NAA/Cr** | 1.29 ± 0.14, n=38 | 1.31 ± 0.10, n=33 | 1.24 ± 0.11, n=24 | 1.33 ± 0.08, n=31 | 1.27 ± 0.09, n=25 | 1.32 ± 0.09, n=26 |
| **Myo-inositol/Cr** | 0.65 ± 0.09, n=38 | 0.66 ± 0.08, n=33 | 0.61 ± 0.10, n=24 | 0.66 ± 0.11, n=31 | 0.64 ± 0.06, n=25 | 0.66 ± 0.09, n=26 |
| **Choline/Cr** | 0.30 ± 0.03, n=38 | 0.31 ± 0.03, n=33 | 0.30 ± 0.03, n=24 | 0.31 ± 0.03, n=31 | 0.30 ± 0.03, n=25 | 0.31 ± 0.03, n=26 |
| **PCr+Cr^B^** | 5.56 ± 0.71, n=38 | 5.58 ± 0.32, n=33 | 5.74 ± 0.36, n=24 | 5.45 ± 0.31, n=31 | 5.60 ± 0.31, n=25 | 5.53 ± 0.30, n=26 |
| **Grey matter (%)** | 19.1 ± 6.8, n=38 | 19.4 ± 4.5, n=33 | 21.3 ± 5.9, n=24 | 18.6 ± 5.7, n=30 | 17.3 ± 5.2, n=25 | 20.0 ± 5.3, n=26 |
| **White matter (%)** | 80.1 ± 7.7, n=38 | 80.5 ± 4.6, n=33 | 78.7 ± 6.1, n=24 | 81.3 ± 5.8, n=30 | 82.6 ± 5.2, n=25 | 79.9 ± 5.3, n=26 |
| ***ACC^C^*** |  |  |  |  |  |  |
| **Glutamate/Cr** | 1.51 ± 0.14, n=37 | 1.55 ± 0.10, n=36 | 1.49 ± 0.14, n=27 | 1.53 ± 0.09, n=32 | 1.49 ± 0.10, n=26 | 1.53 ± 0.10, n=31 |
| **Glx/Cr** | 2.01 ± 0.22, n=37 | 2.03 ± 0.18, n=36 | 1.99 ± 0.26, n=27 | 2.02 ± 0.13, n=32 | 1.98 ± 0.17, n=26 | 2.04 ± 0.15, n=31 |
| **Gln/Cr** | 0.52 ± 0.11, n=32 | 0.52 ± 0.08, n=27 | 0.54 ± 0.11, n=22 | 0.50 ± 0.07, n=29 | 0.53 ± 0.08, n=21 | 0.51 ± 0.08, n=29 |
| **NAA/Cr** | 1.22 ± 0.06, n=37 | 1.26 ± 0.06, n=36 | 1.22 ± 0.06, n=27 | 1.26 ± 0.07, n=32 | 1.23 ± 0.06, n=26 | 1.28 ± 0.09, n=31 |
| **Myo-inositol/Cr** | 0.82 ± 0.06, n=37 | 0.85 ± 0.07, n=36 | 0.82 ± 0.07, n=27 | 0.85 ± 0.08, n=32 | 0.83 ± 0.08, n=26 | 0.87 ± 0.07, n=31 |
| **Choline/Cr** | 0.27 ± 0.03, n=37 | 0.29 ± 0.03, n=36 | 0.27 ± 0.03, n=27 | 0.30 ± 0.03, n=32 | 0.28 ± 0.02, n=26 | 0.30 ± 0.03, n=31 |
| **PCr+Cr^B^** | 5.36 ± 0.23, n=37 | 5.28 ± 0.25, n=36 | 5.38 ± 0.25, n=27 | 5.26 ± 0.25, n=32 | 5.34 ± 0.26, n=26 | 5.23 ± 0.25, n=31 |
| **Grey matter (%)** | 67.6 ± 6.4, n=37 | 68.0 ± 3.8, n=36 | 66.9 ± 5.8, n=27 | 68.0 ± 6.2, n=31 | 66.8 ± 5.0, n=26 | 68.6 ± 5.5, n=31 |
| **White matter (%)** | 14.2 ± 4.2, n=37 | 12.9 ± 2.6, n=36 | 14.1 ± 3.4, n=27 | 13.4 ± 2.7, n=31 | 13.8 ± 2.4, n=26 | 13.5 ± 2.7, n=31 |
| ***ACC^D^*** |  |  |  |  |  |  |
| **GABA/Cr** | 0.101 ± 0.014, n=28 | 0.111 ± 0.011, n=32 | 0.102 ± 0.011, n=20 | 0.109 ± 0.009, n=32 | 0.109 ± 0.013, n=24 | 0.113 ± 0.018, n=29 |
| **Glx/Cr** | 0.107 ± 0.015, n=28 | 0.118 ± 0.010, n=32 | 0.115 ± 0.015, n=20 | 0.114 ± 0.010, n=32 | 0.116 ± 0.014, n=24 | 0.117 ± 0.010, n=29 |
| **PCr+Cr^B^** | 10.96 ± 0.80, n=28 | 10.91 ± 0.65, n=32 | 10.93 ± 0.67, n=20 | 10.84 ± 0.73, n=32 | 10.71 ± 0.57, n=24 | 10.75 ± 0.60, n=29 |
| **Grey matter (%)** | 51.2 ± 4.7, n=28 | 53.2 ± 3.2, n=32 | 51.5 ± 4.2, n=20 | 52.7 ± 4.9, n=31 | 52.7 ± 3.5, n=24 | 53.2 ± 4.4, n=29 |
| **White matter (%)** | 32.3 ± 4.1, n=28 | 30.5 ± 3.0, n=32 | 31.1 ± 3.6, n=20 | 31.3 ± 3.5, n=31 | 30.1 ± 3.1, n=24 | 31.2 ± 3.6, n=29 |

Abbreviations: ACC: Anterior cingulate cortex; FEP: First episode psychosis; HCs: Healthy controls; Glx: glutamate+glutamine; Gln: Glutamine; NAA: N-acetyl aspartate; PCr+Cr: Total creatine (phosphocreatine+creatine); GABA: Gamma-aminobutyric acid. A: n states the number of analyzed spectra. B: Water-scaled value of PCr+Cr provided by LCModel and Gannet and used as reference in metabolite/Cr values. C: Metabolites in a 2.0x2.0x2.0 cm voxel. D: Metabolites in a 3.0x3.0x3.0 cm voxel (details provided in the patients and methods section).

**Table S9. Metabolites in institutional units in ACC and left thalamus at all assessments in first-episode psychosis patients and healthy controls**

|  | **Baseline** | | **6 weeks** | | **26 weeks** | |
| --- | --- | --- | --- | --- | --- | --- |
|  | **FEP** | **HCs** | **FEP** | **HCs** | **FEP** | **HCs** |
| ***Thalamus*** | **Mean ± SD, n^A^** | **Mean ± SD, n^A^** | **Mean ± SD, n^A^** | **Mean ± SD, n^A^**^,^**^E^** | **Mean ± SD, n^A^** | **Mean ± SD, n^A^** |
| **Glutamate IU** | 7.12 ± 0.85, n=38 | 6.80 ± 0.83, n=32 | 6.89 ± 0.79, n=24 | 6.68 ± 0.74, n=30 | 6.76 ± 0.82, n=25 | 7.06 ± 0.73, n=26 |
| **Glx IU** | 9.99 ± 1.50, n=38 | 9.87 ± 1.29, n=33 | 9.75 ± 1.42, n=24 | 9.78 ± 1.10, n=30 | 9.74 ± 1.40, n=25 | 10.09 ± 1.15, n=26 |
| **NAA IU** | 7.13 ± 0.65, n=38 | 7.30 ± 0.52, n=33 | 7.11 ± 0.46, n=24 | 7.24 ± 0.42, n=30 | 7.13 ± 0.38, n=25 | 7.31 ± 0.34, n=26 |
| **Myo-inositol IU** | 3.57 ± 0.50, n=38 | 3.70 ± 0.49, n=33 | 3.51 ± 0.55, n=24 | 3.56 ± 0.62, n=30 | 3.57 ± 0.29, n=25 | 3.65 ± 0.51, n=26 |
| **Choline IU** | 1.66 ± 0.20, n=38 | 1.73 ± 0.15, n=33 | 1.69 ± 0.17, n=24 | 1.70 ± 0.16, n=30 | 1.71 ± 0.16, n=25 | 1.72 ± 0.14, n=26 |
| **PCr+Cr IU** | 5.63 ± 0.66, n=38 | 5.59 ± 0.32, n=33 | 5.74 ± 0.36, n=24 | 5.46 ± 0.31, n=30 | 5.61 ± 0.31, n=25 | 5.54 ± 0.30, n=26 |
| **Grey matter (%)** | 19.1 ± 6.8, n=38 | 19.4 ± 4.5, n=33 | 21.3 ± 5.9, n=24 | 18.6 ± 5.7, n=30 | 17.3 ± 5.2, n=25 | 20.0 ± 5.3, n=26 |
| **White matter (%)** | 80.1 ± 7.7, n=38 | 80.5 ± 4.6, n=33 | 78.7 ± 6.1, n=24 | 81.3 ± 5.8, n=30 | 82.6 ± 5.2, n=25 | 79.9 ± 5.3, n=26 |
| ***ACC^C^*** |  |  |  |  |  |  |
| **Glutamate IU** | 10.94 ± 1.46, n=37 | 11.19 ± 1.00, n=36 | 11.03 ± 1.56, n=27 | 10.98 ± 1.35, n=31 | 10.90 ± 0.88, n=26 | 10.75 ± 0.88, n=31 |
| **Glx IU** | 14.56 ± 2.07, n=37 | 14.66 ± 1.52, n=36 | 14.72 ± 2.44, n=27 | 14.55 ± 1.84, n=31 | 14.54 ± 1.42, n=26 | 14.28 ± 1.31, n=31 |
| **Glutamine IU** | 3.83 ± 0.72, n=32 | 3.79 ± 0.59, n=27 | 3.99 ± 0.80, n=22 | 3.66 ± 0.61, n=28 | 3.87 ± 0.62, n=21 | 3.60 ± 0.57, n=29 |
| **NAA IU** | 8.87 ± 0.98, n=37 | 9.09 ± 0.61, n=36 | 9.01 ± 0.81, n=27 | 9.07 ± 1.01, n=31 | 9.02 ± 0.80, n=26 | 8.96 ± 0.77, n=31 |
| **Myo-inositol IU** | 5.98 ± 0.76, n=37 | 6.12 ± 0.57, n=36 | 6.05 ± 0.78, n=27 | 6.15 ± 0.91, n=31 | 6.11 ± 0.67, n=26 | 6.14 ± 0.70, n=31 |
| **Choline IU** | 1.99 ± 0.27, n=37 | 2.11 ± 0.23, n=36 | 1.99 ± 0.27, n=27 | 2.13 ± 0.28, n=31 | 2.09 ± 0.24, n=26 | 2.11 ± 0.26, n=31 |
| **PCr+Cr IU** | 7.26 ± 0.74, n=37 | 7.24 ± 0.50, n=36 | 7.37 ± 0.68, n=27 | 7.20 ± 0.70, n=31 | 7.35 ± 0.62, n=26 | 7.03 ± 0.62, n=31 |
| **Grey matter (%)** | 67.6 ± 6.4, n=37 | 68.0 ± 3.8, n=36 | 66.9 ± 5.8, n=27 | 68.0 ± 6.2, n=31 | 66.8 ± 5.0, n=26 | 68.6 ± 5.5, n=31 |
| **White matter (%)** | 14.2 ± 4.2, n=37 | 12.9 ± 2.6, n=36 | 14.1 ± 3.4, n=27 | 13.4 ± 2.7, n=31 | 13.8 ± 2.4, n=26 | 13.5 ± 2.7, n=31 |
| ***ACC^D^*** |  |  |  |  |  |  |
| **GABA IU** | 2.32 ± 0.39, n=27 | 2.50 ± 0.28, n=32 | 2.34 ± 0.27, n=20 | 2.43 ± 0.25, n=31 | 2.44 ± 0.31, n=24 | 2.47 ± 0.42, n=29 |
| **Glx IU** | 10.64 ± 1.63, n=27 | 11.25 ± 0.87, n=32 | 11.27 ± 1.30, n=20 | 10.87 ± 1.40, n=31 | 11.06 ± 1.24, n=24 | 10.95 ± 1.17, n=29 |
| **PCr+Cr IU** | 14.54 ± 1.19, n=27 | 14.23 ± 1.06, n=32 | 14.52 ± 1.00, n=20 | 14.11 ± 1.29, n=31 | 14.18 ± 0.81, n=24 | 13.87 ± 1.12, n=29 |
| **Grey matter (%)** | 51.2 ± 4.7, n=28 | 53.2 ± 3.2, n=32 | 51.5 ± 4.2, n=20 | 52.7 ± 4.9, n=31 | 52.7 ± 3.5, n=24 | 53.2 ± 4.4, n=29 |
| **White matter (%)** | 32.3 ± 4.1, n=28 | 30.5 ± 3.0, n=32 | 31.1 ± 3.6, n=20 | 31.3 ± 3.5, n=31 | 30.1 ± 3.1, n=24 | 31.2 ± 3.6, n=29 |

Table S9 shows metabolite levels in institutional units calculated with the formula provided in the supplementary methods.

Abbreviations: ACC: Anterior cingulate cortex; FEP: First episode psychosis; HCs: Healthy controls; IU: institutional units; Glx: glutamate+glutamine; NAA: N-acetyl aspartate; PCr+Cr: Total creatine (phosphocreatine+creatine); GABA: Gamma-aminobutyric acid. A: n states the number of analyzed spectra. B: Water-scaled value of PCr+Cr provided by LCModel and Gannet and used as reference in metabolite/Cr values. C: Metabolites in a 2.0x2.0x2.0 cm voxel. D: Metabolites in a 3.0x3.0x3.0 cm voxel (details provided in the patients and methods section). E: The segmentation of grey and white matter could not be done for 1 HC.

**Table S10. Metabolites scaled to water in ACC and left thalamus at all assessments in first-episode psychosis patients and healthy controls**

|  | **Baseline** | | **6 weeks** | | **26 weeks** | |
| --- | --- | --- | --- | --- | --- | --- |
|  | **FEP** | **HCs** | **FEP** | **HCs** | **FEP** | **HCs** |
| ***Thalamus*** | **Mean ± SD, n^A^** | **Mean ± SD, n^A^** | **Mean ± SD, n^A^** | **Mean ± SD, n^A^** | **Mean ± SD, n^A^** | **Mean ± SD, n^A^** |
| **Glutamate/H_2_0** | 7.04 ± 0.87, n=38 | 6.79 ± 0.82, n=32 | 6.89 ± 0.77, n=24 | 6.70 ± 0.76, n=31 | 6.75 ± 0.81, n=25 | 7.04 ± 0.73, n=26 |
| **Glx/H_2_0** | 9.88 ± 1.55, n=38 | 9.86 ± 1.28, n=33 | 9.74 ± 1.39, n=24 | 9.79 ± 1.10, n=31 | 9.73 ± 1.40, n=25 | 10.07 ± 1.14, n=26 |
| **NAA/H_2_0** | 7.04 ± 0.60, n=38 | 7.29 ± 0.51, n=33 | 7.11 ± 0.45, n=24 | 7.22 ± 0.41, n=31 | 7.13 ± 0.38, n=25 | 7.30 ± 0.34, n=26 |
| **Myo-inositol/H_2_0** | 3.53 ± 0.53, n=38 | 3.70 ± 0.48, n=33 | 3.51 ± 0.54, n=24 | 3.57 ± 0.61, n=31 | 3.57 ± 0.29, n=25 | 3.64 ± 0.51, n=26 |
| **Choline/H_2_0** | 1.64 ± 0.21, n=38 | 1.73 ± 0.15, n=33 | 1.70 ± 0.18, n=24 | 1.69 ± 0.16, n=31 | 1.71 ± 0.16, n=25 | 1.72 ± 0.14, n=26 |
| **PCr+Cr/H_2_0** | 5.56 ± 0.71, n=38 | 5.58 ± 0.32, n=33 | 5.74 ± 0.36, n=24 | 5.45 ± 0.31, n=31 | 5.60 ± 0.31, n=25 | 5.53 ± 0.30, n=26 |
| ***ACC^C^*** |  |  |  |  |  |  |
| **Glutamate/H_2_0** | 8.07 ± 0.78, n=37 | 8.17 ± 0.52, n=36 | 8.02 ± 0.81, n=27 | 8.02 ± 0.50, n=32 | 7.93 ± 0.54, n=26 | 8.00 ± 0.36, n=31 |
| **Glx/H_2_0** | 10.73 ± 1.17, n=37 | 10.69 ± 0.91, n=36 | 10.70 ± 1.48, n=27 | 10.61 ± 0.71, n=32 | 10.56 ± 0.84, n=26 | 10.63 ± 0.60, n=31 |
| **Glutamine/H_2_0** | 2.78 ± 0.55, n=32 | 2.77 ± 0.41, n=27 | 2.88 ± 0.61, n=22 | 2.63 ± 0.37, n=29 | 2.79 ± 0.39, n=21 | 2.67 ± 0.37, n=29 |
| **NAA/H_2_0** | 6.54 ± 0.29, n=37 | 6.63 ± 0.25, n=36 | 6.57 ± 0.34, n=27 | 6.62 ± 0.26, n=32 | 6.55 ± 0.23, n=26 | 6.67 ± 0.37, n=31 |
| **Myo-inositol/H_2_0** | 4.41 ± 0.34, n=37 | 4.47 ± 0.34, n=36 | 4.40 ± 0.36, n=27 | 4.48 ± 0.37, n=32 | 4.43 ± 0.33, n=26 | 4.56 ± 0.31, n=31 |
| **Choline/H_2_0** | 1.47 ± 0.16, n=37 | 1.54 ± 0.12, n=36 | 1.45 ± 0.15, n=27 | 1.55 ± 0.14, n=32 | 1.52 ± 0.12, n=26 | 1.57 ± 0.12, n=31 |
| **PCr+Cr/H_2_0** | 5.36 ± 0.23, n=37 | 5.28 ± 0.25, n=36 | 5.38 ± 0.25, n=27 | 5.26 ± 0.25, n=32 | 5.34 ± 0.26, n=26 | 5.23 ± 0.25, n=31 |
| ***ACC^D^*** |  |  |  |  |  |  |
| **GABA/H_2_0** | 1.76 ± 0.26, n=27 | 1.91 ± 0.20, n=32 | 1.76 ± 0.17, n=20 | 1.86 ± 0.14, n=32 | 1.85 ± 0.23, n=24 | 1.91 ± 0.31, n=29 |
| **Glx/H_2_0** | 8.08 ± 1.08, n=27 | 8.63 ± 0.66, n=32 | 8.49 ± 1.03, n=20 | 8.36 ± 0.80, n=32 | 8.34 ± 0.82, n=24 | 8.49 ± 0.77, n=29 |
| **PCr+Cr/H_2_0** | 11.07 ± 0.58, n=27 | 10.91 ± 0.65, n=32 | 10.93 ± 0.67, n=20 | 10.84 ± 0.73, n=32 | 10.71 ± 0.57, n=24 | 10.75 ± 0.60, n=29 |

Table S10 shows metabolite levels scaled to water as reported in the output from LCModel and Gannet.

Abbreviations: ACC: Anterior cingulate cortex; FEP: First episode psychosis; HCs: Healthy controls; Glx: glutamate+glutamine; NAA: N-acetyl aspartate; PCr+Cr: Total creatine (phosphocreatine+creatine); GABA: Gamma-aminobutyric acid. A: n states the number of analyzed spectra. B: Water-scaled value of PCr+Cr provided by LCModel and Gannet and used as reference in metabolite/Cr values. C: Metabolites in a 2.0x2.0x2.0 cm voxel. D: Metabolites in a 3.0x3.0x3.0 cm voxel (details provided in the patients and methods section).

**Table S11. Baseline neurometabolite levels in institutional units in responders and non-responders after 6 and 26 weeks compared with HCs**

|  | **Non-responders**  **6 weeks** | **Responders**  **6 weeks** | **HCs** | **P_6 weeks_^D^**  **NR vs HCs** | **P_6 weeks_^D^**  **R vs HCs** | **Non-responders**  **26 weeks** | **Responders**  **26 weeks** | **HCs** | **P_26 weeks_^D^**  **NR vs HCs** | **P_26 weeks_^D^**  **R vs HCs** |
| --- | --- | --- | --- | --- | --- | --- | --- | --- | --- | --- |
| ***Thalamus***  ***Baseline levels*** | **Mean ± SD, (n^A^)** | **Mean ± SD, (n^A^)** | **Mean ± SD, (n^A^)** |  |  | **Mean ± SD, (n^A^)** | **Mean ± SD, (n^A^)** | **Mean ± SD, (n^A^)** |  |  |
| **Glutamate IU** | 7.06 ± 0.70, (18) | 7.12 ± 0.70, (11) | 6.81 ± 0.84, (31) | P=0.21 | P=0.30 | 7.39 ± 0.82, (15) | 7.23 ± 0.83, (9) | 6.81 ± 0.87, (28) | P=0.033 | P=0.28 |
| **Glx IU** | 9.58 ± 1.05, (18) | 9.97 ± 1.30, (11) | 9.90 ± 1.30, (32) | P=0.42 | P=0.87 | 10.55 ± 1.83, (15) | 10.09 ± 0.73, (9) | 9.93 ± 1.32, (29) | P=0.18 | P=0.86 |
| **NAA IU** | 7.15 ± 0.68, (18) | 7.09 ± 0.50, (11) | 7.30 ± 0.52, (32) | P=0.38 | P=0.29 | 7.17 ± 0.65, (15) | 7.15 ± 0.76, (9) | 7.32 ± 0.54, (29) | P=0.47 | P=0.51 |
| **Myo-inositol IU** | 3.55 ± 0.43, (18) | 3.49 ± 0.59, (11) | 3.71 ± 0.49, (32) | P=0.14 | P=0.27 | 3.56 ± 0.54, (15) | 3.64 ± 0.59, (9) | 3.74 ± 0.48, (29) | P=0.23 | P=0.69 |
| **Choline IU** | 1.62 ± 0.21, (18) | 1.69 ± 0.15, (11) | 1.74 ± 0.14, (32) | P=0.017 | P=0.41 | 1.68 ± 0.19, (15) | 1.61 ± 0.20, (9) | 1.74 ± 0.14, (29) | P=0.29 | P=0.04 |
| **PCr+Cr IU** | 5.41 ± 0.53, (18) | 5.67 ± 0.40, (11) | 5.61 ±0.31, (32) | P=0.05 | P=0.50 | 5.59 ± 0.41, (15) | 5.48 ± 0.76, (9) | 5.59 ± 0.32, (29) | P=0.99 | P=0.61 |
| **Grey matter (%)** | 20.7 ± 6.5, (18) | 17.8 ± 7.2, (11) | 19.3 ± 4,6 (32) | P=0.33 | P=0.37 | 19.5 ± 9.0, (15) | 18.4 ± 5.8, (9) | 19.4 ± 4.6, (29) | P=0.87 | P=0.65 |
| **White matter (%)** | 78.3 ± 8.9, (18) | 82.1 ± 7.3, (11) | 80.6 ± 4.6, (32) | P=0.18 | P=0.44 | 78.6 ± 10.7, (15) | 81.5 ± 5.8, (9) | 80.5 ± 4.6, (29) | P=0.36 | P=0.68 |
| ***ACC^B^  Baseline levels*** | |  |  |  |  |  |  |  |  |  |
| **Glutamate IU** | 10.80 ± 1.84, (17) | 10.76 ± 1.12, (11) | 11.15 ± 0.98, (35) | P=0.60 | P=0.23 | 11.28 ± 1.42, (15) | 10.57 ± 1.61, (8) | 11.21 ± 1.00, (32) | P=0.88 | P=0.15 |
| **Glx IU** | 14.29 ± 2.46, (17) | 14.33 ± 1.90, (11) | 14.61 ± 1.52, (35) | P=0.91 | P=0.39 | 15.05 ± 1.66, (15) | 14.09 ± 2.46, (8) | 14.64 ± 1.58, (32) | P=0.46 | P=0.31 |
| **Gln IU** | 3.67 ± 0.83, (15) | 4.04 ± 0.70, (8) | 3.80 ± 0.61, (26) | P=0.85 | P=0.56 | 3.77 ± 0.76, (15) | 3.74 ± 0.86, (7) | 3.81 ± 0.62, (23) | P=0.86 | P=0.83 |
| **NAA IU** | 8.92 ± 1.10, (17) | 8.50 ± 0.84, (11) | 9.07 ± 0.61, (35) | P=0.63 | P=0.04 | 9.02 ± 0.99, (15) | 8.47 ± 1.35, (8) | 9.12 ± 0.60, (32) | P=0.66 | P=0.05 |
| **Myo-inositol IU** | 6.04 ± 0.83, (17) | 5.77 ± 0.74, (11) | 6.11 ± 0.57, (35) | P=0.96 | P=0.14 | 6.02 ± 0.81, (15) | 5.91 ± 0.97, (8) | 6.18 ± 0.54, (32) | P=0.47 | P=0.34 |
| **Choline IU** | 1.98 ± 0.32, (17) | 1.93 ± 0.23, (11) | 2.10 ± 0.24, (35) | P=0.27 | P=0.03 | 2.01 ± 0.29, (15) | 1.94 ± 0.34, (8) | 2.12 ± 0.24, (32) | P=0.17 | P=0.07 |
| **PCr+Cr** | 7.30 ± 0.69, (17) | 6.98 ± 0.81, (11) | 7.22 ± 0.50, (35) | P=0.44 | P=0.21 | 7.44 ± 0.68, (15) | 6.87 ± 1.00, (8) | 7.26 ± 0.50, (32) | P=0.36 | P=0.12 |
| **Grey matter (%)** | 67.5 ± 6.8, (17) | 69.4 ± 4.5, (11) | 68.0 ± 3.9, (35) | P=0.73 | P=0.42 | 65.2 ± 5.9, (15) | 70.9 ± 8.0, (8) | 67.3 ± 3.4, (32) | P=0.18 | P=0.09 |
| **White matter (%)** | 13.4 ± 3.5, (17) | 15.1 ± 5.5, (11) | 13.0 ± 2.6, (35) | P=0.85 | P=0.03 | 14.5 ± 5.0, (15) | 14.0 ± 4.0, (8) | 13.0 ± 2.7, (32) | P=0.15 | P=0.36 |
| ***ACC^C^Baseline levels*** | |  |  |  |  |  |  |  |  |  |
| **GABA IU** | 2.39 ± 0.37, (11) | 2.23 ± 0.46, (8) | 2.49 ± 0.28, (31) | P=0.36 | P=0.05 | 2.37 ± 0.40, (8) | 2.42 ± 0.35, (5) | 2.51 ± 0.27, (28) | P=0.21 | P=0.61 |
| **Glx IU** | 10.43 ± 1.78, (11) | 10.22 ± 1.28, (8) | 11.23 ± 0.87, (31) | P=0.13 | P=0.02 | 11.37 ± 0.87, (8) | 10.59 ± 0.99, (5) | 11.27 ± 0.86, (28) | P=0.73 | P=0.08 |
| **PCr+Cr** | 14.81 ± 0.92, (11) | 13.35 ± 1.66, (8) | 14.22 ± 1.07, (31) | P=0.12 | P=0.36 | 14.90 ± 1.11, (8) | 13.86 ± 1.23, (5) | 14.27 ± 1.09, (28) | P=0.15 | P=0.47 |
| **Grey matter (%)** | 51.0 ± 3.1, (11) | 53.7 ± 3.6, (9) | 53.1 ± 3.2 , (31) | P=0.07 | P=0.82 | 48.9 ± 5.4, (8) | 53.3 ± 3.8, (6) | 52.7 ± 2.9, (28) | P=0.02 | P=0.99 |
| **White matter (%)** | 31.9 ± 2.4, (11) | 32.5 ± 5.1, (9) | 30.5 ± 3.1, (31) | P=0.38 | P=0.14 | 32.9 ± 3.8, (8) | 32.3 ± 5.2, (6) | 30.6 ± 3.2, (28) | P=0.11 | P=0.32 |

Table S11 shows baseline levels of metabolites in institutional units calculated with the formula provided in the supplementary methods. Abbreviations: IU: institutional units; NR: Non-responders; R: Responders; HCs: Healthy controls; ACC: Anterior cingulate cortex; Glx: Glutamate+glutamine; Gln: glutamine; NAA: N-acetyl aspartate; PCr+Cr: Total creatine (phosphocreatine+creatine); GABA: Gamma-aminobutyric acid. A: N is the number of spectra analyzed. B: Metabolites in a 2.0x2.0x2.0 cm voxel. C: Metabolites in a 3.0x3.0x3.0 cm voxel (details provided in the patients and methods section). D: Adjusted for age and sex.

**Table S12. Baseline neurometabolite levels scaled to water in responders and non-responders after 6 and 26 weeks compared with HCs**

|  | **Non-responders**  **6 weeks** | **Responders**  **6 weeks** | **HCs** | **P_6 weeks_^D^**  **NR vs HCs** | **P_6 weeks_^D^**  **R vs HCs** | **Non-responders**  **26 weeks** | **Responders**  **26 weeks** | **HCs** | **P_26 weeks_^D^**  **NR vs HCs** | **P_26 weeks_^D^**  **R vs HCs** |
| --- | --- | --- | --- | --- | --- | --- | --- | --- | --- | --- |
| ***Thalamus***  ***Baseline levels*** | **Mean ± SD, (n^A^)** | **Mean ± SD, (n^A^)** | **Mean ± SD, (n^A^)** |  |  | **Mean ± SD, (n^A^)** | **Mean ± SD, (n^A^)** | **Mean ± SD, (n^A^)** |  |  |
| **Glutamate/H_2_0** | 6.95 ± 0.74, (18) | 7.11 ± 0.67, (11) | 6.80 ± 0.84, (31) | P=0.43 | P=0.30 | 7.19 ± 0.92, (15) | 7.23 ± 0.83, (9) | 6.80 ± 0.86, (28) | P=0.16 | P=0.29 |
| **Glx/H_2_0** | 9.43 ± 1.18, (18) | 9.95 ± 1.29, (11) | 9.88 ± 1.29, (32) | P=0.25 | P=0.87 | 10.28 ± 1.99, (15) | 10.08 ± 0.74, (9) | 9.91 ± 1.31, (29) | P=0.44 | P=0.86 |
| **NAA/H_2_0** | 7.02 ± 0.66, (18) | 7.08 ± 0.48, (11) | 7.29 ± 0.52, (32) | P=0.12 | P=0.28 | 6.96 ± 0.52, (15) | 7.14 ± 0.75, (9) | 7.31 ± 0.53, (29) | P=0.06 | P=0.50 |
| **Myo-inositol/H_2_0** | 3.50 ± 0.51, (18) | 3.49 ± 0.59, (11) | 3.71 ± 0.49, (32) | P=0.09 | P=0.29 | 3.47 ± 0.63, (15) | 3.63 ± 0.59, (9) | 3.74 ± 0.48, (29) | P=0.11 | P=0.71 |
| **Choline/H_2_0** | 1.60 ± 0.24, (18) | 1.69 ± 0.15, (11) | 1.74 ± 0.14, (32) | P=0.008 | P=0.44 | 1.64 ± 0.23, (15) | 1.61 ± 0.20, (9) | 1.73 ± 0.14, (29) | P=0.09 | P=0.05 |
| **PCr+Cr/H_2_0** | 5.33 ± 0.62, (18) | 5.66 ± 0.40, (11) | 5.60 ±0.31, (32) | P=0.02 | P=0.53 | 5.45 ± 0.59, (15) | 5.47 ± 0.76, (9) | 5.58 ± 0.32, (29) | P=0.39 | P=0.66 |
| ***ACC^B^***  ***Baseline levels*** |  |  |  |  |  |  |  |  |  |  |
| **Glutamate/H_2_0** | 7.82 ± 0.94, (17) | 8.32 ± 0.52, (11) | 8.14 ± 0.51, (35) | P=0.14 | P=0.55 | 8.04 ± 0.70, (15) | 8.18 ± 0.67, (8) | 8.11 ± 0.51, (32) | P=0.71 | P=0.84 |
| **Glx/H_2_0** | 10.34 ± 1.25, (17) | 11.07 ± 1.05, (11) | 10.67 ± 0.91, (35) | P=0.57 | P=0.39 | 10.73 ± 0.75, (15) | 10.90 ± 1.33, (8) | 10.59 ± 0.89, (32) | P=0.62 | P=0.47 |
| **Gln/H_2_0** | 2.63 ± 0.52, (15) | 3.04 ± 0.63, (8) | 2.78 ± 0.41, (26) | P=0.70 | P=0.27 | 2.69 ± 0.51, (15) | 2.83 ± 0.72, (7) | 2.75 ± 0.40, (23) | P=0.97 | P=0.67 |
| **NAA/H_2_0** | 6.47 ± 0.34, (17) | 6.57 ± 0.26, (11) | 6.62 ± 0.25, (35) | P=0.01 | P=0.73 | 6.42 ± 0.30, (15) | 6.53 ± 0.22, (8) | 6.60 ± 0.23, (32) | P=0.02 | P=0.48 |
| **Myo-inositol/H_2_0** | 4.39 ± 0.41, (17) | 4.45 ± 0.26, (11) | 4.46 ± 0.34, (35) | P=0.43 | P=0.96 | 4.28 ± 0.36, (15) | 4.57 ± 0.33, (8) | 4.48 ± 0.36, (32) | P=0.09 | P=0.41 |
| **Choline/H_2_0** | 1.44 ± 0.16, (17) | 1.49 ± 0.12, (11) | 1.53 ± 0.12, (35) | P=0.03 | P=0.35 | 1.43 ± 0.16, (15) | 1.49 ± 0.10, (8) | 1.53 ± 0.13, (32) | P=0.01 | P=0.44 |
| **PCr+Cr/H_2_0** | 5.31 ± 0.28, (17) | 5.39 ± 0.19, (11) | 5.28 ± 0.25, (35) | P=0.74 | P=0.16 | 5.31 ± 0.27, (15) | 5.31 ± 0.19, (8) | 5.25 ± 0.24, (32) | P=0.43 | P=0.47 |
| ***ACC^C^***  ***Baseline levels*** |  |  |  |  |  |  |  |  |  |  |
| **GABA/H_2_0** | 1.80 ± 0.19, (11) | 1.78 ± 0.40, (8) | 1.91 ± 0.20, (31) | P=0.14 | P=0.18 | 1.75 ± 0.23, (8) | 1.89 ± 0.17, (5) | 1.91 ± 0.19, (28) | P=0.04 | P=0.89 |
| **Glx/H_2_0** | 7.86 ± 1.12, (11) | 8.14 ± 1.12, (8) | 8.62 ± 0.67, (31) | P=0.03 | P=0.13 | 8.44 ± 0.60, (8) | 8.29 ± 0.75, (5) | 8.58 ± 0.64, (28) | P=0.66 | P=0.28 |
| **PCr+Cr/H_2_0** | 11.21 ± 0.51, (11) | 10.97 ± 0.70, (8) | 10.90 ± 0.66, (31) | P=0.26 | P=0.62 | 11.05 ± 0.52, (8) | 10.83 ± 0.60, (5) | 10.85 ± 0.63, (28) | P=0.33 | P=0.96 |

Table S12 shows metabolite levels scaled to water as reported in the output from LCModel and Gannet.

Abbreviations: NR: Non-responders; R: Responders; HCs: Healthy controls; ACC: Anterior cingulate cortex; Glx: Glutamate+glutamine; Gln: glutamine; NAA: N-acetyl aspartate; PCr+Cr: Total creatine (phosphocreatine+creatine); GABA: Gamma-aminobutyric acid. A: N is the number of spectra analyzed. B: Metabolites in a 2.0x2.0x2.0 cm voxel. C: Metabolites in a 3.0x3.0x3.0 cm voxel (details provided in the patients and methods section). D: Adjusted for age and sex.
